# When Tech Meets Touch: Multistakeholder Perspectives and Implementation Strategies for eHealth in Chronic Kidney Disease: A Systematic Review Using Computational Linguistics

**DOI:** 10.1155/jonm/7612011

**Published:** 2026-05-13

**Authors:** Xutong Zheng, Liuxuan Xie, Yingshuo Liu, Qiuying Yu, Han Zhang, Xiao Zhang, Aiping Wang

**Affiliations:** ^1^ Department of Public Service, The First Affiliated Hospital of China Medical University, Shenyang, China, cmu.edu.cn

**Keywords:** CFIR–ERIC mapping, chronic kidney disease, digital health, e-Health, implementation science, sentiment analysis, stakeholder perspectives

## Abstract

**Background:**

Chronic kidney disease heavily burdens patients and health systems. Digital health interventions offer significant potential but face implementation challenges driven by stakeholders’ emotional and practical experiences. This review synthesizes these affective dimensions to inform clinical practice.

**Methods:**

A qualitative systematic review was conducted across six databases for studies published through April 2025. Methodological quality was rigorously appraised. We integrated Python‐based computational sentiment analysis to quantify stakeholder emotional polarity (positive, negative, neutral) with thematic analysis. Identified barriers were mapped to established implementation frameworks to select expert‐validated implementation strategies.

**Results:**

Twenty‐three qualitative studies were included, revealing five domains: accessibility, communication, workflow, empowerment, and clinical effectiveness. Patients praised digital empowerment but highlighted socioeconomic access barriers. Nurses valued workflow efficiencies but reported role ambiguity. Clinicians expressed deep skepticism toward remote clinical effectiveness due to diagnostic limitations. To resolve these tensions, prioritized implementation strategies include identifying clinical champions, promoting intervention adaptability, and systematically assessing organizational readiness before deployment.

**Conclusion:**

Stakeholder emotions critically dictate digital health adoption. For clinical practice, these findings emphasize moving beyond generic deployments. Healthcare systems must address clinician diagnostic concerns via hybrid care models, resolve nurse workflow ambiguities through targeted training, and provide low‐cost devices to bridge patient equity gaps. Tailoring solutions to these psychosocial needs is essential for successful integration into routine kidney care.

## 1. Introduction

Chronic kidney disease (CKD) represents a mounting global public health crisis, affecting approximately 10% of the global population, with projections indicating it will become the fifth leading cause of death by 2040 [1–3]. Nationally and locally, the incidence of progressive CKD and end‐stage renal disease (ESRD) continues to surge, placing an unprecedented and unsustainable strain on healthcare resources [1, 4]. The disease’s trajectory—from early‐stage management to ESRD requiring dialysis or transplantation—imposes multifaceted burdens: patients endure reduced quality of life [5, 6], complex treatment regimens [7, 8], and frequent healthcare interactions [7, 9]; clinicians navigate intricate care coordination [10, 11]; and health systems face escalating costs [12, 13].

To address these profound clinical challenges, modern CKD management has fundamentally shifted toward a multidimensional and multidisciplinary care framework [14, 15]. Optimal patient care now extends far beyond mere pharmacological intervention; it requires seamless collaboration among nephrologists, specialized nurses, dietitians, and social workers to address the complex clinical and psychosocial needs of patients [14, 15]. Within this modern clinical practice standpoint, specific assistive strategies are paramount. For instance, specialized and dedicated care programs tailored for patients starting dialysis have proven essential in reducing early mortality, alleviating patient anxiety, and ensuring a smoother transition to ESRD treatments [16, 17]. Furthermore, as the CKD demographic ages, routine frailty assessments using validated tools have become a cornerstone of clinical practice, guiding individualized treatment pathways and mitigating adverse outcomes [18, 19]. Central to the success of these multidisciplinary models are the evolving competencies of nephrology nurses. Nephrology nurses act as the critical linchpin in this ecosystem, taking on advanced roles in coordinating assistive practice, conducting ongoing symptom assessments, and delivering continuous patient education [15].

It is specifically within this intricate, multidisciplinary clinical context that digital health interventions (DHIs)—including telehealth, electronic patient‐reported outcome measures (ePROMs), remote monitoring, and mobile self‐management tools—offer transformative potential. By enabling remote consultations, real‐time symptom tracking, and personalized education, DHIs promise to enhance the accessibility [20, 21], patient empowerment [22, 23], and clinical efficiency required to sustain modern CKD care [20, 24]. For example, the implementation of remote monitoring and digital self‐management programs has been shown to significantly improve patient outcomes by facilitating the early detection of fluid overload to reduce hospitalization rates, stabilizing renal function decline through improved medication adherence, and alleviating depressive symptoms by enhancing patient activation and self‐efficacy [25].

However, the successful implementation of DHIs hinges critically on the *diverse perceptions* of stakeholders integral to the care ecosystem [26, 27]. Additionally, these perspectives are often emotionally charged—ranging from enthusiasm about time savings to frustration with technical barriers or distrust in virtual assessments. Understanding not only what stakeholders think about these interventions but also how they feel about them—their attitudes, concerns, satisfaction levels, and emotional responses—is crucial for successful implementation. Traditional qualitative syntheses, though valuable, typically focus on thematic content without systematically capturing the *affective valence* (positive, negative, neutral sentiments) underlying stakeholder narratives. This gap is consequential: Sentiment polarity directly influences engagement, adherence, and equity. Sentiment analysis provides a systematic approach to examining these emotional and attitudinal dimensions of stakeholder experiences, offering insights into the underlying factors that drive acceptance or resistance to DHIs [28, 29].

This systematic review bridges this methodological gap by integrating computational sentiment analysis with established qualitative synthesis to decode the emotional dimensions of stakeholder experiences. Therefore, our guiding research question is: What are the affective and practical determinants of digital health implementation in CKD management, and which tailored strategies can address them? To answer this, the primary objective of this study was to identify implementation barriers and facilitators and map them to the Consolidated Framework for Implementation Research (CFIR) to inform the selection of targeted strategies via the Expert Recommendations for Implementing Change (ERIC) framework. The secondary objectives were to (1) quantify sentiment polarity using Valence Aware Dictionary and sEntiment Reasoner (VADER) and (2) generate stakeholder‐specific insights by stratifying these sentiments across groups to reveal how emotional undertones influence adoption.

Unlike conventional content analysis, this dual approach captures not only *what* stakeholders express but how they feel, providing an objective quantification of affective responses that traditionally elude manual thematic coding. To systematically translate these complex emotional and practical insights into actionable solutions, our methodology integrates the CFIR and the ERIC framework. The CFIR provides a robust, multidomain typology (comprising intervention characteristics, outer setting, inner setting, characteristics of individuals, and process) to comprehensively diagnose implementation barriers and facilitators. Subsequently, the ERIC framework offers a validated compilation of 73 specific implementation strategies tailored to address these identified determinants. The combination of CFIR and ERIC has proven highly effective in the wider evidence base; for example, this mapping approach has been successfully utilized to tailor implementation strategies for scaling up telemental health services and to integrate them into routine practice [30, 31], demonstrating its robust utility in bridging the gap between barrier identification and strategic action. These insights directly inform the selection of implementation strategies from the ERIC framework, ensuring solutions address both practical and affective barriers to DHI success in CKD care.

## 2. Methods

### 2.1. Study Design and Reproducibility Statement

**FIGURE 1:**
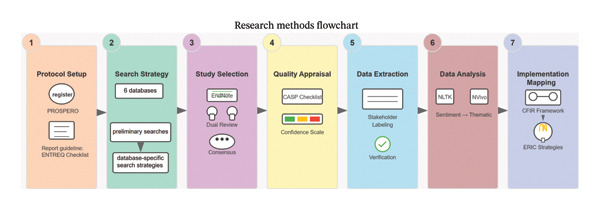
Research flowchart.

To ensure high transparency, scientific consistency, and international reproducibility, this study was designed as a hybrid computational‐qualitative systematic review. The methodology systematically integrates automated sentiment analysis (computational linguistics) with reflexive thematic analysis and deductive implementation framework mapping. To guarantee reproducibility, the entire process strictly adhered to the PRISMA guidelines for systematic reviews and the Enhancing Transparency in Reporting the Synthesis of Qualitative Research (ENTREQ) checklist for synthesizing qualitative research [32]. The whole methodological workflow could be found in Figure 1. As depicted in the flowchart, our approach is structured into three main phases: (1) systematic literature search and selection, (2) hybrid inductive–deductive qualitative synthesis (incorporating both thematic and sentiment analysis), and (3) implementation strategy mapping using the CFIR and ERIC frameworks. A detailed, step‐by‐step explanation of the procedures involved in each of these phases is provided in the subsequent subsections below.

### 2.2. Protocol and Registration

This systematic review protocol was registered with PROSPERO (registering number: CRD42023473931) in October 2023.

### 2.3. Inclusion and Exclusion Criteria

The inclusion and exclusion criteria are presented in Table 1.

**TABLE 1:** Inclusion and exclusion criteria.

Inclusion criteria	Exclusion criteria
Studies must meet ALL of the following: 1. Study Design: • Qualitative or mixed‐methods research with explicit qualitative data collection (e.g., interviews, focus groups, observations) and analysis (e.g., thematic analysis, content analysis, grounded theory, phenomenology); • Must have verbatim quotes supporting identified themes. 2. Population • Adults (≥ 18 years) with CKD (Stages 1–5, including dialysis/transplant) OR • Healthcare professionals (e.g., nephrologists, nurses) OR • Caregivers/family members of CKD patients. 3. Intervention and Focus • Digital health interventions targeting CKD management (e.g., telehealth, ePROMs, mobile apps, remote monitoring, e‐consultations, SMS/text messaging). • Must explore experiences, perceptions, or impacts on health outcomes (e.g., adherence, self‐management, care coordination). 4. Publication Type • Peer‐reviewed, full‐text articles in English. 5. Context • Any geographic setting or healthcare context (e.g., hospitals, community clinics, home‐based care).	Studies excluded if they meet ANY of the following: 1. Design Limitations • Mixed‐methods studies where qualitative data cannot be extracted separately. • Studies without original qualitative data (e.g., reviews, opinion pieces, protocols). 2. Intervention Mismatch • Digital tools used solely for data collection (e.g., EHRs for administrative purposes) without a focus on patient/HCP experiences or health outcomes. 3. Outcome Mismatch • Focuses only on technical feasibility (e.g., app development) without exploring user experiences/health impacts. • Justification: Excludes studies lacking health outcome relevance (e.g., software testing without patient/HCP input).

### 2.4. Search Strategy

The search strategy was executed in three phases. Initially, author X.T. conducted preliminary searches on PubMed and Embase using both controlled vocabulary (MeSH and Emtree) and free‐text terms to generate a broad keyword set, which was then reviewed by the research team. In the second phase, authors X.T. and Y.S. collaborated with a medical librarian to develop and iteratively refine database‐specific search strategies, balancing sensitivity and specificity through adjustments in terminology and pilot screenings [33]. The final search, covering literature up to April 2025, was conducted across PubMed, Embase, Scopus, Web of Science, CINAHL (EBSCO), and APA PsycINFO (EBSCO). In the third phase, reference lists of all included studies were manually screened for additional eligible publications. Full search strategies are detailed in Supporting File 1.

### 2.5. Study Selection

Study selection followed a two‐phase screening process. First, titles, abstracts, and keywords were manually reviewed against predefined eligibility criteria. In the second phase, full‐text articles of potentially relevant studies were assessed for inclusion. Search results were imported into EndNote X9 for deduplication, after which the remaining records were manually screened. To ensure consistency among the research team, a manual calibration exercise was conducted: 50 randomly selected PubMed articles were independently reviewed by all team members, and assessments were verified by the lead author (X.T.) [34]. During both pilot and formal screening, at least two independent reviewers (authors X.T., Y.S., L.X., and Z.X.) assessed each study, with disagreements resolved by discussion and consensus.

### 2.6. Quality Appraisal and Confidence Grading

The methodological quality of included studies was evaluated using the Critical Appraisal Skills Programme (CASP) checklist, which emphasizes key aspects of qualitative research such as rigor, trustworthiness, and reflexivity. To ensure consistency between reviewers, a manual calibration exercise was conducted, with disagreements resolved through team discussions. Interrater reliability was assessed by having two reviewers independently appraise a subset of 5 studies (21.74%). No studies were excluded based solely on quality, recognizing that even lower quality studies can offer valuable insights.

Confidence in the synthesized findings was assessed using the GRADE‐CERQual approach, which evaluates four domains: methodological limitations, coherence, data adequacy, and relevance [35]. Each finding was assigned a confidence level: no or very minor concerns, minor concerns, moderate concerns, or serious concerns. While the first two levels did not lower confidence, moderate or serious concerns led to a downgraded confidence rating.

### 2.7. Data Extraction

Data extraction was conducted systematically by author X.T. using a standardized form to ensure consistency and completeness. Key study details—such as authorship, publication year, location, study objectives, methodology, and participant characteristics—were recorded uniformly across all studies. A separate qualitative extraction form was used to capture core findings and illustrative quotes. For stakeholder‐specific sentiment and thematic analysis, data elements such as study ID, third‐order constructs, and stakeholder identity labels (e.g., patient, clinician) were extracted. Any verbatim quotes without a clear stakeholder label will be excluded. To minimize bias, data were independently reviewed by team members, with all extracted content cross‐verified by a second reviewer (author Y.S.). Any discrepancies were resolved through discussion, involving a third reviewer (author Z.H.) when necessary.

### 2.8. Data Analysis

To analyze opinions from stakeholders like nephrologists and nurses about eHealth interventions, a two‐step process will be used. First, an automated tool was used to classify opinions as positive, negative, or neutral. Then, a manual process was used to identify common themes within these opinions, focusing on each stakeholder group separately. This combined approach was intended to provide a clear and detailed understanding of how different healthcare professionals view eHealth interventions. The analysis utilized the Natural Language Toolkit (NLTK) in Python for sentiment analysis and NVivo 12.0 for thematic analysis, with results synthesized narratively for each stakeholder group and sentiment category.

#### 2.8.1. Data Preparation

Relevant textual data, specifically opinions or statements from stakeholders regarding eHealth interventions, will be extracted from the included studies. Each opinion will be tagged with its corresponding stakeholder group (e.g., nephrologists, nurses) to ensure traceability. The extracted texts will be cleaned and formatted as necessary to prepare them for analysis, ensuring consistency in data input for both NLTK and NVivo.

#### 2.8.2. Sentiment Analysis

Sentiment analysis will be conducted using the VADER tool within the NLTK in Python [28, 29]. VADER is a lexicon and rule‐based sentiment analysis tool designed to assess the emotional tone of text, particularly effective for short and semistructured texts. Each extracted opinion will be processed to generate a compound sentiment score ranging from −1 (*most negative*) to +1 (*most positive*). Based on standard thresholds, opinions will be classified as follows: (1) **Positive**: compound score > 0; (2) **Negative**: compound score < 0; (3) **Neutral**: compound score = 0 [28, 29].

The sentiment classification will be validated through a subset of manual checks to ensure accuracy, particularly given the potentially nuanced nature of stakeholder opinions in qualitative studies. The Python script for sentiment analysis will be developed to process the dataset systematically, outputting a dataset where each opinion is labeled with its sentiment and stakeholder group.

#### 2.8.3. Thematic Analysis

Thematic analysis will follow Braun and Clarke’s six‐phase framework, chosen for its flexibility and depth in exploring stakeholder perspectives [36, 37]. In Phase 1 (Familiarization), researchers will repeatedly read extracted data to gain a comprehensive understanding and identify initial patterns. In Phase 2 (Generating Initial Codes), data will be systematically coded using NVivo 12.0, ensuring full coverage by assigning descriptive labels to all relevant segments. In Phase 3 (Searching for Themes), codes will be organized into themes and subthemes based on conceptual relationships, capturing key viewpoints on eHealth interventions. These themes will then be refined and validated in Phase 4 (Reviewing Themes) to ensure they accurately reflect both coded content and the broader dataset. Phase 5 (Defining and Naming Themes) will involve articulating each theme’s meaning and relevance to the research aims, including sentiments—positive, negative, or neutral—toward specific aspects of eHealth. Finally, in Phase 6 (Producing the Report), themes will be synthesized into a structured narrative incorporating sentiment analysis. This synthesis will highlight stakeholder‐specific patterns—for example, nephrologists linking eHealth with “enhanced patient monitoring,” while nurses may express concerns about “increased workload.” Themes will be stratified by both sentiment and stakeholder group to capture distinct viewpoints and provide actionable insights for implementation. These inductively generated themes and their associated sentiments established the foundational data for the subsequent deductive framework analysis.

### 2.9. Mapping Into CFIR Framework and Selection of ERIC Strategies

To identify effective implementation strategies for eHealth interventions in CKD management, we employed a hybrid inductive–deductive qualitative analysis approach. Following the inductive thematic analysis, we conducted a deductive framework analysis using the original 2009 version of the CFIR. This specific version was selected to ensure compatibility with the validated CFIR–ERIC Matching Tool utilized in our subsequent steps. Specifically, two independent reviewers (X.T. and L.X.) systematically deductively coded the inductively generated themes, barriers, facilitators, and sentiment‐stratified data against the five overarching CFIR domains: intervention characteristics, outer setting, inner setting, characteristics of individuals, and process [38, 39]. For example, themes related to “workflow integration” were deductively mapped to the inner setting construct, while “accessibility” issues were mapped to either intervention characteristics or outer setting, depending on the context. Discrepancies in deductive coding were resolved through discussion with a third reviewer (A.P.). Based on this comprehensive deductive mapping, we used the ERIC tool to select strategies addressing the specific CFIR‐coded barriers.

To ensure rigor and transparency, an audit trail was maintained throughout the process, documenting all decisions and revisions. At least four team members (authors A, B, C, D, and F) cross‐verified ERIC strategy selections, resolving discrepancies through consensus. Additionally, selections were reviewed by experts in CKD, digital health, and implementation science to ensure contextual relevance and feasibility. The strategies were refined iteratively through ongoing feedback and expert input, ensuring adaptability to emerging insights. To systematically prioritize which ERIC strategies would be of most use, we utilized a quantitative aggregation approach derived from the CFIR–ERIC matching tool. For each identified CFIR barrier, the matching tool provides an endorsement percentage representing expert consensus on a strategy’s effectiveness. We calculated a “cumulative percent” by summing these endorsement scores across all the specific CFIR barriers identified in our qualitative synthesis. Specifically, because our qualitative data mapped onto 11 distinct CFIR constructs, and the maximum expert endorsement for a single construct is 100%, the theoretical maximum cumulative score any ERIC strategy could achieve in our study is 1100%. To enhance interpretability for a broader audience outside of implementation science, we also express this metric as an overall proportional agreement (calculated by dividing the cumulative score by the 1100% maximum). Strategies yielding the highest cumulative scores were prioritized for our final recommendations, as this mathematical aggregation indicated their high utility in comprehensively addressing multiple implementation barriers simultaneously. The strategies were refined iteratively through ongoing feedback and expert input, ensuring adaptability to emerging insights.

## 3. Results

### 3.1. Study Selection

**FIGURE 2:**
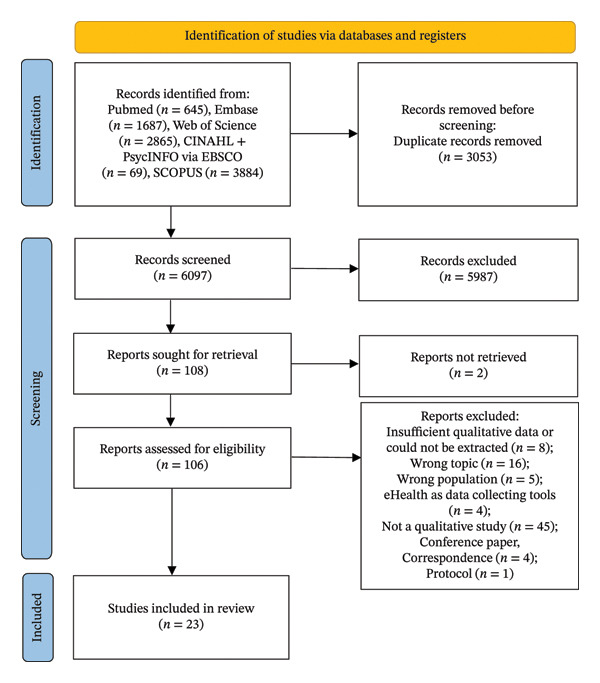
Study selection flow diagram.

The systematic review adhered to the PRISMA 2020 guidelines for study selection. Initial searches across five databases—PubMed (645 records), Embase (1687 records), Web of Science (2865 records), CINAHL and PsycINFO via EBSCO (69 records), and Scopus (3884 records)—yielded 9150 records. Following the removal of 3053 duplicates, 6097 records underwent title and abstract screening, of which 5987 were excluded for not meeting the inclusion criteria. Full‐text retrieval was attempted for 108 records, but 2 were inaccessible, leaving 106 records for full‐text eligibility assessment. Of these, 83 were excluded for failing to meet eligibility criteria. Twenty‐three studies satisfied all inclusion requirements and were included in the final analysis. The complete selection process is illustrated in the PRISMA flow diagram (Figure 2).

### 3.2. Characteristics of Included Results

The systematic review included 23 qualitative studies exploring DHIs for patients with CKD (Table 2), conducted across diverse settings including the USA (*n* = 5), Australia (*n* = 5), Canada (*n* = 3), Denmark (*n* = 3), UK (*n* = 2), New Zealand (*n* = 2), Iran (*n* = 1), Brazil (*n* = 1), and China (*n* = 1). These studies, published between 2017 and 2025, primarily utilized semistructured interviews, focus groups, and thematic analysis to investigate stakeholder perspectives, including those of patients, clinicians, caregivers, and healthcare professionals. Participant groups ranged from 10 to 80 individuals, encompassing CKD patients (Stages 3–5, including dialysis and transplant recipients), clinicians (nephrologists, nurses), and caregivers, with ages spanning 18–100 years and balanced gender representation in most studies. The interventions examined included ePROMs, telehealth (video visits, remote monitoring, telephone coaching), mobile text messaging, and eHealth self‐management tools, such as dietary apps and electronic personal health records (ePHRs).

**TABLE 2:** Characteristics of studies included.

Study ID	Country	Aim	Characteristics of participants	Methodological and sampling approach	Method of data collection and analysis	Key concepts and themes
Aiyegbusi, Olalekan et al., (2019)	UK	To explore the perspectives of patients and clinicians on the use of a renal ePROM	12 patients (5 women, 7 men) with 91.6% (*n* = 11) aged ≥ 50 22 clinicians (14 women, 8 men)	Ethnography and descriptive methodology combination of purposive and snowball sampling	Semistructured face‐to‐face or telephone interviews; focus group discussion thematic data analysis coding	Electronic patient‐reported outcome measures (ePROMs); general opinions of PROMs; potential benefits and applications of an ePROM system; practical considerations for the implementation of ePROMs; concerns, barriers, and facilitators
Aminu K. Bello et al., 2017	Canada	We assessed stakeholder perceptions on the use of an electronic consultation system (e‐Consult) to improve the delivery of kidney care in Alberta. We aim to identify acceptability, barriers and facilitators to the use of an e‐Consult system for ambulatory kidney care delivery.	72 participants in two broad stakeholder categories: patients (including patients’ relatives) and providers (including primary care physicians, nephrologists, other care providers and policymakers); 36 provider and 36 patient; 21 male providers; 15 female providers; 17 male patients; 19 female patients	General qualitative study; purposive sampling	Focus groups; audio‐recorded and transcribed verbatim; Semistructured interview; Picker institute model, thematic analysis	The key barriers identified were length of time required for referring physicians to complete the e‐Consult due to lack of integration with current electronic medical records, and concerns that increased numbers of requests might overwhelm nephrologists and lead to a delayed response or an unsustainable system. The key facilitators identified were potential improvement of care coordination, dissemination of best practice through an educational platform, comprehensive data to make decisions without the need for face‐to‐face consultation, timely feedback to primary care providers, timeliness/reduced delays for patients’ rapid triage and identification of cases needing urgent care and improved access to information to facilitate decision‐making in patient care.
Ginger Chu et al., 2024	Australia	To explore patients’ and carers’ perspectives as they transition to telehealth‐assisted home visits and identify the factors influencing their engagement in this modality.	Twenty‐three participants (21 home dialysis patients and two carers) 7 hemodialysis patients, 14 Peritoneal dialysis patients; patients aged 49–81; 13 male patients and 8 female patients;	General qualitative study; convenience sample	Semistructured interviews by phone; framework analysis; COM‐B model	Capability: (a) skills to conduct assessments and (b) usability in a diverse group. Opportunity: (a) social opportunity and support, (b) clinician sentiment, and (c) technological issues. Motivation: (a) suboptimal practice (barrier), (b) entrenched views of technology (barrier), and (c) benefits of telehealth (facilitator),
Jessica Dawson et al., 2021	Australia	Dietary requirements for people on hemodialysis are complex and often poorly adhered to. Mobile phone text messaging offers a simple strategy to enhance current nutritional care. KIDNEYTEXT was a 6‐month pilot randomized controlled trial that evaluated the feasibility and impact of mobile phone text messages to alter participants’ dietary behaviors. The aim of this study was to elicit the perspectives of people on hemodialysis regarding acceptability of mobile phone text messages targeting dietary behaviors.	25 adult patients (2 in‐center dialysis patients, 17 community dialysis patients, and 6 home dialysis patients); aged 40–79; 16 male, 9 female	Grounded theory; Purposively sampled	Semistructured interviews (in‐person or over the phone); thematic analysis; audio‐recorded and transcribed verbatim	building awareness (reinforcement of information, simple and comprehensible, guiding choices, accessible information enhancing motivation, gaining skills in management), valuing care (boosting self‐esteem, in‐person care bolstered by reminders), activating change (adjusting lifestyle, gaining control of electrolytes and fluid, striving to improve overall health), waning attention and motivation (lack of personalization limiting change, maintaining the status quo, reverting back to old habits)
Ditchburn and Marshall et al., 2017	UK	The purpose of this paper is to present the perspectives of the renal care team members using the renal telemedicine service to understand the perceived benefits and issues with the service.	10 members of renal care team (2 renal specialists, 1 matron, 2 renal nurses, 1 business manager, 1 renal technical services manager, 2 IT technicians and 1 hardware maintenance technician)	General qualitative study; the stakeholder empowered adoption model	Semistructured interviews; thematic analysis; an inductive approach; double‐coded	Six major themes: (i) positive feedback from patients, (ii) quicker way to solve problems, (iii) more efficient use of staff time, (iv) reduced travel, (v) peace of mind, and (vi) sense of job satisfaction
Maoliosa Donald et al., 2022	Canada	There is limited research of electronic tools for self‐management for patients with chronic kidney disease (CKD). We sought to evaluate participant engagement, perceived self‐efficacy and website usage in a preliminary evaluation of My Kidneys My Health, a patient‐facing eHealth tool in Canada.	15 participants, self‐reported a diagnosis of CKD, not on kidney replacement therapy and access to the Internet using a computer, tablet or mobile device; aged ≥ 18; 8 male, 7 female	Mixed‐methods study (interpretive qualitative research); convenience sample	Semistructured interviews by phone; content analysis	Acceptability of website: ease of use, adoption of website strategies: self‐management integration, implementation of MKMH: determinants of effective implementation
Cyd Eaton et al., 2020	USA	In this multiphase investigation, we developed and tested a theoretically informed text messaging intervention based on the COM‐B model, a well‐established health intervention framework stating that capability, opportunity, and motivation interactively modify health behaviors, to improve participants’ antihypertensive medication adherence in a pilot randomized controlled trial. Qualitative data on user experiences were obtained.	18 AYA with CKD (Reminder + COM‐B Message Intervention); mean age 17; 7 female, 11 male	General qualitative study; purposively sampled	Semistructured interviews by phone; content analysis	
Suad Esayed et al., 2025	USA	We aimed to understand patient experiences and preferences regarding telemedicine video visits and highlight insights to advance adopting hybrid telemedicine/in‐person transplant care.	20 adult (≥ 18 years old) kidney transplant recipients (follow‐up at ≥ 12 months post‐transplant via telemedicine at a tertiary transplant center); aged 52–72; 10 female, 10 male	Inductive thematic qualitative study; purposive sampling;	In‐depth semistructured interviews; audio‐ and video‐recorded; thematic analysis;	Theme 1: Reducing Travel Time, Theme 2: Minimizing Financial Burden, Theme 3: Engaging Patients Within Their Comfort Space, Theme 4: Establishing Rapport With Patients, Theme 5: Limitations of the Virtual Physical Exam, Theme 6: Enhancing Access to Transplant Providers, Theme 7: Lowering Risk of Communicable Diseases
Birgith Engelst et al., 2023	Denmark	This study aimed to explore the patients’ experiences using PROs in remote care and how this mode of follow‐up may enhance patient engagement.	15 patients (chronic kidney disease experienced with PRO‐based remote follow‐up in 3 renal outpatient clinics in the Central Denmark Region); 9 male, 6 female; Aged 50–89;	Ethnography and interpretive qualitative research; purposive sampling	Semistructured interviews (face‐to‐face and by phone); thematic analysis	Facilitators to patient engagement. Barriers to patient engagement (PRO improves communication, PRO increases disease knowledge, PRO‐based remote follow‐up induces flexibility, clinician feedback increases feelings of security, PRO prompts clinical actions). Barriers to patient engagement (lack of feedback induces a feeling of being unsupported, self‐assessment requires disease knowledge, PRO competes with biomedical data, PRO‐based remote follow‐up challenges patient‐physician relation)
Trenton M. Haltom, 2024	USA	This study describes patients’ perspectives on the use of telemedicine during in‐center hemodialysis.	32 patients (14 male and 18 female) from underserved populations (older, less educated, unemployed, persons of color) (aged 18 or older)	General qualitative study; convenience sampling	Telephone semistructured interviews; an iterative process and thematic analysis.	1. Adapting to telemedicine. 2. Ensuring availability of the physician. 3. Safeguarding against infection. 4. Straining communication and physical interactions. 5. Maintaining privacy. 6. Supporting confidence in telemedicine
Jaimon T. Kelly, 2019	Australia	To evaluate the feasibility and acceptability of a personalized telehealth intervention to support dietary self‐management in adults with Stages 3–4 chronic kidney disease (CKD).	80 patients: Intervention group (*n* = 41): 26 male aged 62.0 ± 12.0. Control group (*n* = 39): 25 male aged 61.1 ± 13.3	Mixed‐methods study; consecutive sampling	Questionnaire and semistructured interviews; descriptive statistics and qualitative content analysis	1. Characteristics of participants. 2. Reach and retention. 3. Intervention delivery. 4. Intervention adherence
Keren Ladin, 2021	USA	To identify patient, care partner, and nephrologists’ perceptions of the patient‐centeredness, benefits, drawbacks of telehealth compared to in‐person visits.	60 interviews (19 [32%] clinicians; 30 [50%] patients, and 11 [18%] care partners); of the patients, 13 (43%) were non‐Hispanic Black participants, 20 (67%) were women, and 22 (73%) were 75 years or older. Sixteen clinicians (84%) were nephrologists	General qualitative study; purposive sampling	Semistructured interview guides based on literature review and clinical experience; thematic analysis	1. Variable Quality of Care. 2. Patient Engagement With Clinicians. 3. Loss of Interpersonal Connection and Mistrust. 4. Difficulty of Breaking Bad News. 5. Disparities Accessing Telehealth
Charlotte Nielsen, 2020	Denmark	To explore patients’ and healthcare professionals’ experiences of using a telehealth solution developed to improve follow‐up after kidney transplantation.	16 patients, 4 nurses (between 10 and over 30 years of experience) and 16 doctors (9 consultants and 7 junior doctors)	An explorative qualitative study with a phenomenological–hermeneutic approach; purposive sampling	Semistructured interviews with patients and a focus group; based on Ricoeur’s theory on narrative and interpretation, structural analysis using NVivo 11 software	1. Challenging conditions for training sessions. 2. Telehealth improves patient reflection and collaboration. 3. Telehealth gives patients a voice in consultations.
Abdelhady Osman, 2024	Canada	To understand patients with CKD views on telemedicine clinics during the pandemic compared to traditional in‐person clinics.	12 English‐speaking patients with CKD (6 male, 6 female) aged 53–79, (mean 66), with at least one in‐person nephrology visit before March 15, 2020, and one Telemedicine appointment after March 30, 2020.	Qualitative descriptive study; purposeful sampling	Semistructured interviews; directed content analysis	Key themes: (1) convenience; building connection and trust; necessity of in‐person care; role of family or caregivers; (5) preferences for clinic types
Raquel Scofano, 2022	Brazil	To evaluate whether telemedicine provided through telemonitoring can improve the ongoing relationship between the doctor, the nurse and the patient.	17 patients (aged 60–100, mean 80; most male), white, had completed higher education, 3.2 years on HHD; 12 nurses (aged 38–48, mean 43; 76% women), 70% white, a mean duration of professional experience of 5.5 years	General qualitative research; purposeful sampling	Semistructured interviews; focus group technique; thematic–categorical content analysis	A total of 17 patients and 12 nurses were included. We observed that the patients and nurses had positive experiences with telemonitoring and highlighted feelings of being cared for and improved confidence, although they indicated that telemonitoring does not replace face‐to‐face visits.
Hongxia Shen, 2022	China	To inform the adaptation of a tailored eHealth self‐management intervention for patients with CKD in China based on the Dutch Medical Dashboard (MD) intervention, we examined the perceptions, attitudes and needs of Chinese patients with CKD and healthcare professionals (HCPs) toward eHealth‐based (self‐management) interventions in general and the Dutch MD intervention in specific.	11 patients with CKD (aged 18–61, mean 38.9; 6 female); 9 patients with CKD (aged 18–61 > 61, mean 43.3; 5 female); 10 HCP (aged 21–50, mean 33; 9 female)	Interpretive qualitative study cross‐sectional qualitative study; purposive sampling convenience sampling	Semistructured interviews and focus group discussions; qualitative analysis	Three themes emerged: (1) experience with eHealth in CKD (self‐management), (2) needs for supporting CKD self‐management with the use of eHealth, and (3) adaptation and implementation of the Dutch MD intervention in China.
Louise Birk Suder et al., 2023	Denmark	To investigate patients’ needs in relation to a dietary app for patients with CKD, patients’ and health professionals’ immediate responses to a prototype dietary app and their suggestions for improvement and further development of the app.	Seven patients, aged 46–82, 2 females and 5 males, Stages 4 or 5 CKD and not be on dialysis; a nephrologist, a head nurse, three nurses in charge, two nurses with special interest in nutrition and two nurses responsible for education and development.	Analytical qualitative research	Semistructured individual interviews and focus groups; thematic analysis, interpretive description, code separately	Key Themes: 1. the dietary challenges for patients with CKD and their need for nutritional counselling 2. patients’ and health professionals’ responses to the prototype app, as well as suggestions for improvements and further development
Esmaeel Toni et al., 2021	Iran	To understand users’ needs and requirements in chronic kidney disease (CKD) care to consider in the design of an ePHR to facilitate its implementation, adoption, and use.	15 CKD patients, aged 21–79, 9 males and 6 females, 10 nephrology nurses, aged 22–47.	General Qualitative Research; Purposive sampling	Semistructured, face‐to‐face, one‐on‐one, and focus group interviews; thematic analysis, systematically code, transcribe verbatim	Key Themes: Factors that influence ePHR adoption by CKD patients (Personal factors, Environmental factors, Technological factors, Chronic disease factors)
Alexi Vahlkamp et al., 2024	USA	To describe the feasibility of engaging older Veterans with advanced CKD in GOC conversations via telehealth	11 patients, aged ≥ 70, 7 in‐person/phone,4 via telehealth, CKD Stages 4 or 5; 4 palliative care clinicians, 2 physicians and 2 nurse practitioners	Interpretive qualitative research; purposive sampling	Semistructured interview; inductive, rapid analytic approach and the constant comparison method; transcribe verbatim	Key Themes: 1. engaging veterans with advanced CKD in GOC conversations was influenced by a number of factors (i.e., key barriers and facilitators); 2. engaging veterans in GOC conversations affected their perception of their care.
Viecelli et al., 2022	Australia	To explore patient and clinician perspectives on ePROMs monitoring with feedback to clinicians.	41 participants (12 patients, 13 nephrologists, 16 dialysis nurses); 12 patients (5 females, 7 males; aged 39–88); 16 dialysis nurses (11 females, 5 males, with 8.8 years of HD experience); 13 nephrologists (6 females,7 males, with 20 years of HD experience)	Analytical qualitative research; purposive sampling	Face‐to‐face semistructured interviews and focus group discussions; thematic analysis	Enabling efficient, systematic, and multidisciplinary patient‐centered care; 2. experiencing limited data and options for symptom management; 3. requiring familiarity with technology and processes; 4. identifying barriers and competing priorities.
Walker et al., 2020	New Zealand	To describe clinicians’ perspectives and experiences of remote monitoring in caring for patients on PD.	13 registered nurses and 12 nephrologists or nephrologists in training (all had experience with remote patient monitoring for APD ranging from less than 1 year to one and a half years)	Descriptive qualitative research; purposive and snowballing sampling	Semistructured interviews; thematic analysis	1. Promoting and maintaining PD; 2. enabling data‐driven decisions; 3. establishing boundaries for use; 4. enhancing patient‐focused care
Walker et al., 2020	New Zealand	To describe patients’ and caregivers’ expectations and experiences of remote monitoring for APD.	34 participants (27 patients and 7 caregivers, aged 31–76, over half were married, 15 were in full‐ or part‐time employment and 16 had vocational or university qualifications.)	Analytical qualitative research; purposive sampling	Semistructured face‐to‐face interviews; Thematic analysis	Reducing patient burden; 2. Strengthening partnerships in care; 3. Improving access to treatment; 4. Preserving quality patient–provider interactions
Warner et al., 2019	Australia	To describe the acceptability and experiences of a telehealth coaching intervention that utilized telephone calls and tailored text messages to improve diet quality in patients with stage 3‐4 CKD.	21 patients (14 males, 7 females; aged 22–78)	Analytical qualitative research; consecutive sampling	Semistructured interview (six telephone calls, one every 2 weeks and tailored text messages, one to four per week); The principles of grounded theory and thematic analysis	1. Valuing relationships; 2. Appreciating convenience; 3. Empowered with Actionable knowledge; 4. Increasing diet consciousness; 5. Making sense of complexity

### 3.3. Quality Appraisal and Confidence Grading Results

The methodological quality of the included studies was systematically appraised using the CASP Checklist (Supporting file 2). This 10‐item tool evaluates the rigor, credibility, and overall relevance of qualitative research, with each item rated as “Yes,” “Partial Yes,” “Unclear,” or “No.” Overall, the studies demonstrated a commendably high level of methodological quality, with a substantial proportion (43.5%, 10 out of 23 studies) achieving a perfect score of 100% “Yes” on all appraisal criteria. Indeed, the criteria related to “Clear statement of aims,” “Appropriate qualitative methodology,” “Research design appropriate to address the aims,” “Data collection,” and “Value of study” all received a universal 100% “Yes” rating across all included studies, underscoring a strong foundation in these fundamental research components.

While the majority of studies exhibited robust methodological practices, some areas were identified where clarity or completeness could be enhanced. Specifically, the “Relationship between researcher and participants” criterion had the lowest “Yes” percentage at 73.91%, followed by “Data analysis rigor” at 82.61%. Despite these minor variations, the overall assessment confirms that the body of evidence included in this review is generally of high methodological quality, providing a strong basis for the synthesis of findings.

Based on the CERQual assessment, confidence in the review findings varied across the main concepts (Table 3). High confidence was assigned to the evidence concerning communication dynamics, workflow integration, patient empowerment, and clinical effectiveness, reflecting no or very minor concerns across methodological limitations, coherence, adequacy, and relevance domains for these concepts. In contrast, confidence in the evidence for accessibility was assessed as moderate, primarily due to moderate concerns regarding the adequacy of data supporting this finding, despite no or very minor concerns in the other domains. Details could be found in Supporting file 3.

**TABLE 3:** CERQual table.

Main category of concept	Number of studies contributing to the review finding	Methodological limitations	Coherence	Adequacy	Relevance	CERQual assessment of confidence in the evidence
Accessibility	14 studies	No or very minor concerns	No or very minor concerns	Moderate concerns	No or very minor concerns	Moderate confidence

Communication dynamics	15 studies	No or very minor concerns	No or very minor concerns	No or very minor concerns	No or very minor concerns	High confidence

Workflow integration	15 studies	Minor concerns	No or very minor concerns	No or very minor concerns	No or very minor concerns	High confidence

Patient empowerment	13 studies	Minor concerns	No or very minor concerns	No or very minor concerns	No or very minor concerns	High confidence

Clinical effectiveness	20 studies	Minor concerns	No or very minor concerns	No or very minor concerns	No or very minor concerns	High confidence

### 3.4. Sentiment Analysis Results

As illustrated in Figure 3, the sentiment of all stakeholder subgroups was dominated by positive emotion. This section presents the integrated findings of our dual‐method approach. The five overarching domains detailed below (Sections 3.4.1–3.4.5)—accessibility, communication dynamics, workflow integration, patient empowerment, and clinical effectiveness—represent the primary themes generated from our inductive thematic analysis. We utilized these inductive themes as the structural framework to contextualize the sentiment analysis. Within each theme, findings are stratified by stakeholder group. For each group, we first report the quantitative sentiment polarity distribution (positive, negative, neutral) and subsequently integrate the qualitative inductive data to explain the specific drivers behind these emotional responses. Overall, as illustrated in Figure 3, the sentiment across most stakeholder subgroups was dominated by positive emotion, though significant, nuanced tensions exist. To provide a clear overview of how the inductive qualitative findings map onto the affective sentiments across different stakeholders, a comprehensive ecosystem summary is provided in Table 4.

FIGURE 3Scatter plot of sentiment.

**Figure:**
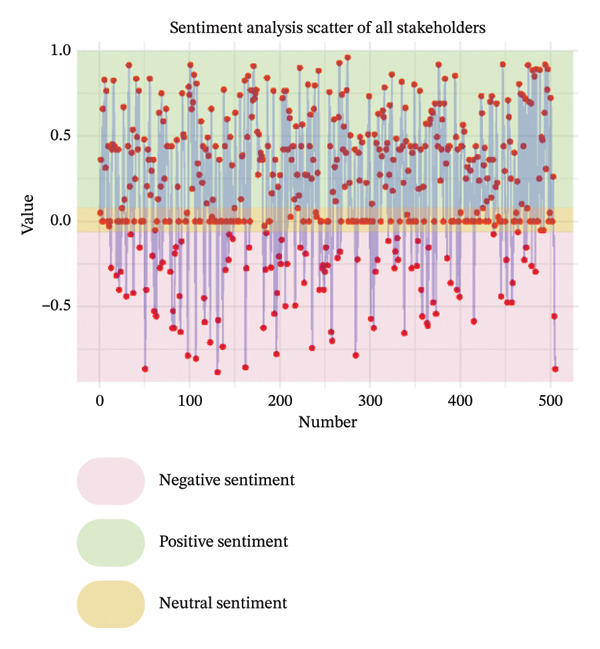
(a)

**Figure:**
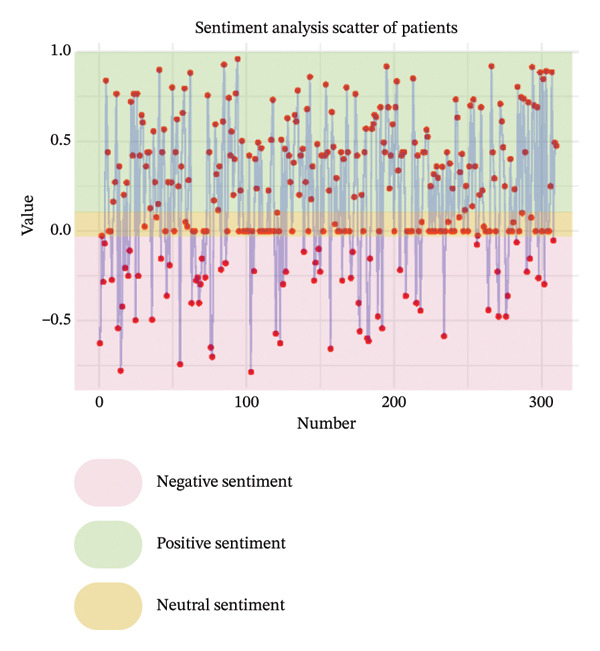
(b)

**Figure:**
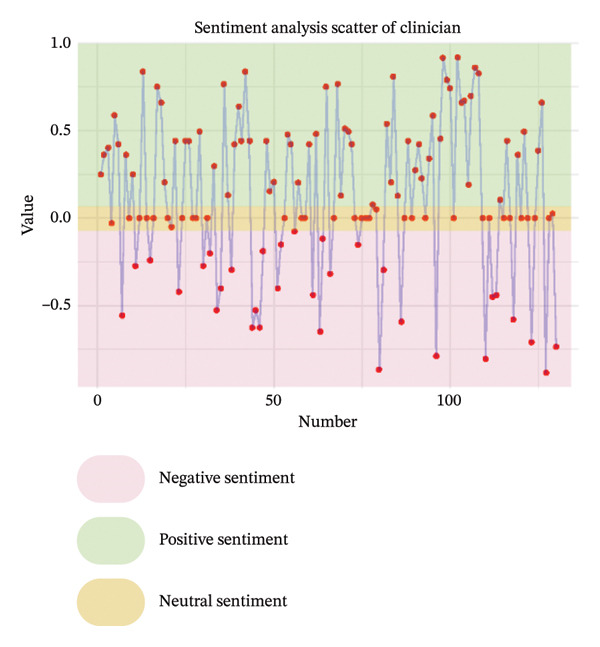
(c)

**Figure:**
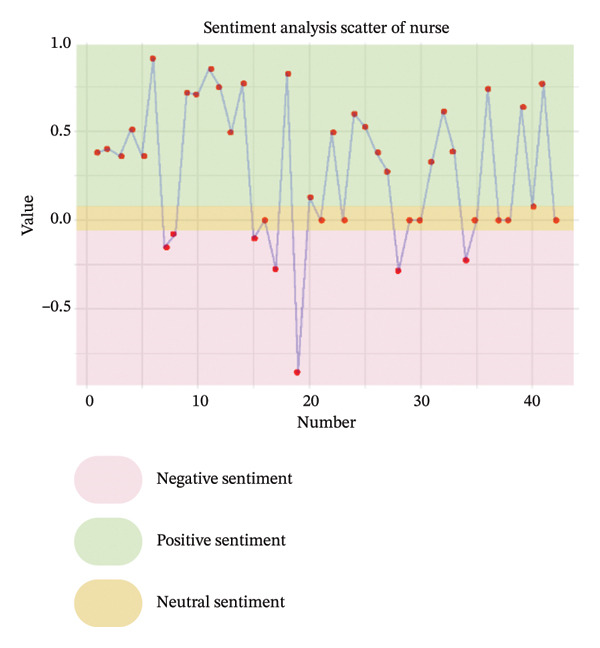
(d)

**Figure:**
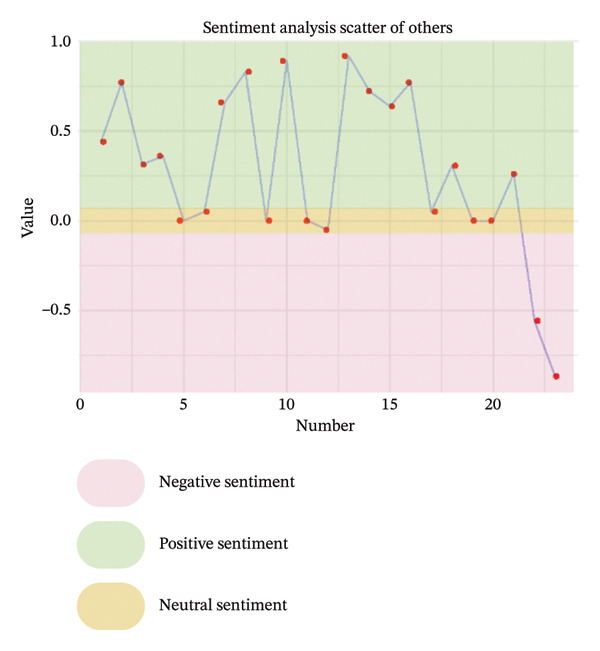
(e)

**TABLE 4:** Ecosystem of themes and sentiment integration.

Inductive theme	Stakeholder	Sentiment polarity	Key inductive qualitative findings (drivers of sentiment)
1. Accessibility	Patients	Positive	Valued flexibility, time savings, reduced travel burden, and inclusion of remote family.
		Negative	Frustrated by tech/socioeconomic barriers, digital literacy gaps, physical strain, and hidden costs.
		Neutral	Displayed pragmatic acceptance; balanced digital convenience against reliability.
	Clinicians	Positive	Appreciated transcending geographic barriers, scheduling flexibility, and seeing home environments.
		Negative	Highlighted unresolved tech failures and systemic inequities marginalizing vulnerable groups.
		Neutral	Objectively noted telehealth magnifies preexisting disparities; success depends on hybrid models.
	Nurses	Positive	Telemedicine complemented in‐person visits; streamlined information retrieval via ePHRs.
		Negative	Highlighted critical infrastructure gaps and geographic/financial burdens impeding access.
		Neutral	Acknowledged transitional benefits (e.g., simpler access to historical data) rather than transformative change.

2. Communication dynamics	Patients	Positive	Found telehealth convenient; PROMs helped organize thoughts for focused conversations.
		Negative	Perceived loss of personal connection/nonverbal cues; frustrated by untimely feedback.
		Neutral	Described factual experiences with variable communication quality across platforms.
	Clinicians	Positive	Enhanced collaborative care (family involvement) and deepened empathy via remote insights.
		Negative	Feared loss of therapeutic touch, eye contact, and emotional context risking superficial interactions.
		Neutral	Highlighted pragmatic adaptations (e.g., EMR summaries, managing environmental distractions).
	Nurses	Positive	Noted tech democratized communication, mitigated hierarchical gaps, and built bidirectional trust.
		Negative/Neutral	*(Minimal presence)* Acknowledged minor limitations in maintaining completely seamless digital rapport.

3. Workflow integration	Patients	Positive	Appreciated convenience, cost‐effective management, and integration of ePROMs.
		Negative	Frustrated by tech issues, slow info flow, and clinicians failing to utilize submitted data.
		Neutral	Offered practical suggestions (e.g., reminders) and noted varying comfort with communication modes.
	Clinicians	Positive	Endorsed efficiency gains, focused consultations, and faster data tracking via automation.
		Negative	Experienced alert fatigue in EHRs, workload constraints, and feared unsustainable referral volumes.
		Neutral	Stressed technical compatibility, context‐dependent utility, and need for standardized team understanding.
	Nurses	Positive	Reported significant time savings (e.g., shorter calls) and workflow flexibility via multitasking.
		Negative	Highlighted inadequate training and role ambiguity creating tension over complex decisions.
		Neutral	Addressed the “time‐efficiency paradox” (long‐term benefits vs. immediate time constraints for adoption).

4. Patient empowerment	Patients	Positive	Empowered by actionable knowledge, simplified guidelines, and personalized features alleviating isolation.
		Negative	Anxious about unreliable online info, irrelevant standardized content, and tools requiring high baseline knowledge.
		Neutral	Displayed pragmatic acceptance; stressed tools cannot replace human dietitians/expertise.
	Clinicians	Positive	Provided psychological reassurance, continuous oversight, and literacy‐adapted educational content.
		Negative	Feared ePROMs creating unrealistic outcome expectations and medicalizing normal stressful experiences.
		Neutral	Questioned the burden on severely ill patients; empowerment strategies require careful calibration.
	Nurses	Positive	Viewed tools as enabling “shared care,” giving power to patients, and reassuring anxious home‐care patients.
		Negative	Stressed privacy control is non‐negotiable; lack of patient authority undermines trust and agency.
		Neutral	Noted structural limitations (e.g., visual impairment, low literacy) preventing engagement for some.

5. Clinical effectiveness	Patients	Positive	Satisfied with telehealth for stable periods, minor issues, and early detection of distress via ePROMs.
		Negative	Doubted diagnostic accuracy for issues needing visual/physical checks; preferred in‐person for changes.
		Neutral	Acknowledged context‐dependent utility; advocated for hybrid models combining virtual/physical care.
	Clinicians	Positive	Recognized systematic symptom detection for earlier issue identification and care coordination.
		Negative	Expressed fundamental concerns about compromised diagnostic rigor, safety risks, and litigation.
		Neutral	Pointed out clinical ambiguity due to safety concerns, though remote monitoring aided adherence.
	Nurses	Positive	Leveraged real‐time visual assessments for quick problem resolution and proactive care (preventing readmissions).
		Negative	Raised data reliability concerns; flawed data could compromise problem‐solving.
		Neutral	Pragmatically noted data timeliness issues (delayed symptom surveys diminish ability to act).

#### 3.4.1. Accessibility

From Figure 4(a), patients expressed moderately positive sentiment (43% positive, 35% negative, 22% neutral), with flexibility and integration into daily life—particularly time savings and reconciling healthcare with work—being dominant positive themes [40–44]. Geographic accessibility significantly benefited rural patients or those unable to drive by reducing travel burdens, while remote participation enhanced family inclusion and urgent care access, fostering a sense of regained autonomy described as feeling “in charge of their own life” [41, 43–48]. Conversely, negative sentiment centered on persistent technological and socioeconomic barriers, including digital literacy gaps and inadequate infrastructure, which excluded some patients [40, 41, 46, 47, 49, 50]. The physical strain of travel remained a significant burden, exacerbated by health limitations, while hidden costs like parking fees, missed wages, and technical disruptions eroded trust [41, 42, 50–52]. Critically, negative comments highlighted systemic inequities, noting telehealth inadvertently marginalized elderly patients and those with “low interest in technology,” with unstable living conditions compounding access challenges. Neutral perspectives reflected pragmatic acceptance, acknowledging telehealth’s efficiency but noting limitations such as phone visits lacking visual cues; access was often conditional, balancing convenience against reliability and personal capability [41, 43, 46, 48, 52].

FIGURE 4Nightingale rose chart staked chart.

**Figure:**
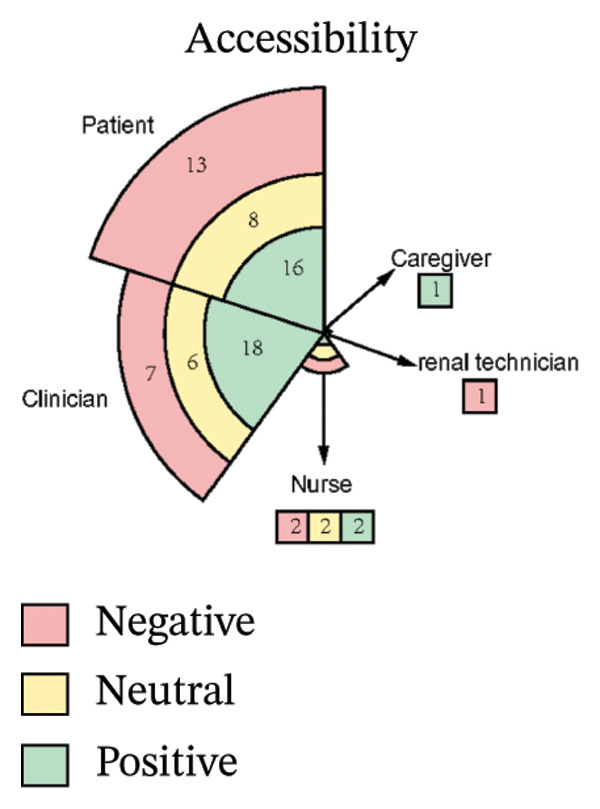
(a)

**Figure:**
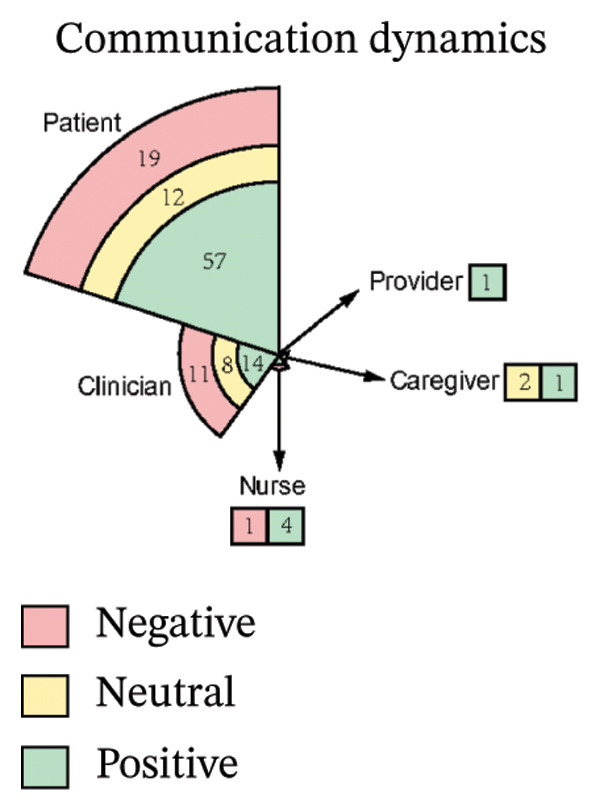
(b)

**Figure:**
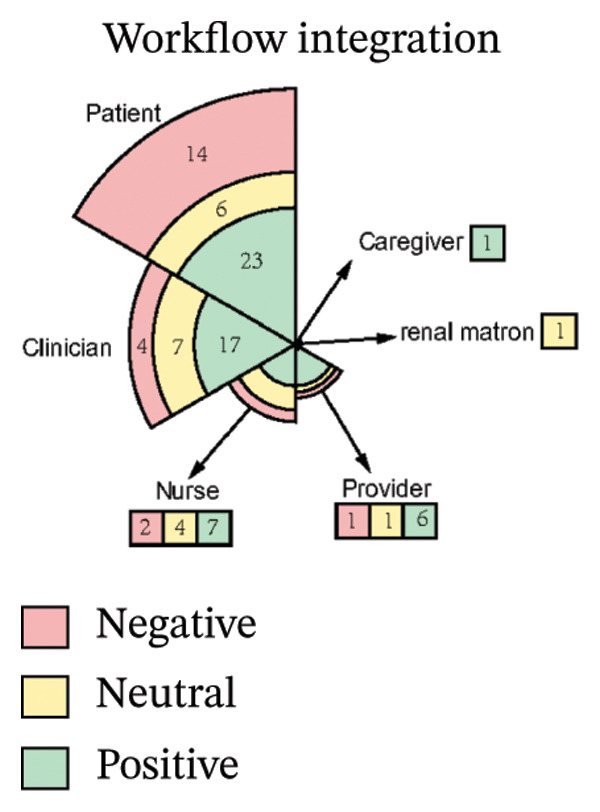
(c)

**Figure:**
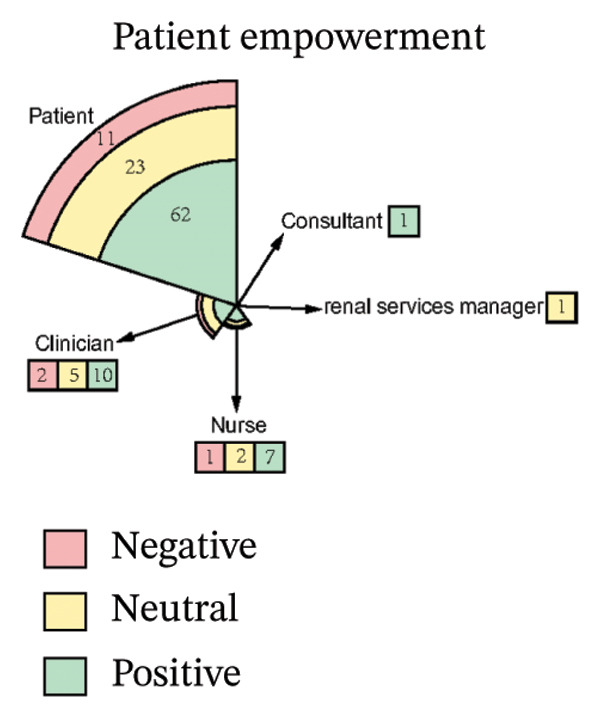
(d)

**Figure:**
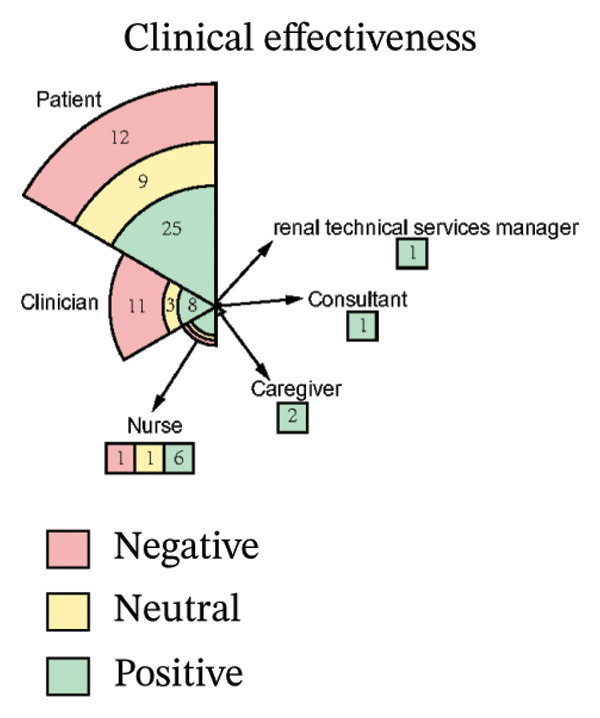
(e)

Clinicians demonstrated predominantly positive sentiment (58% positive, 23% negative, 19% neutral), emphasizing telehealth’s ability to transcend geographical barriers for remote, housebound, or mobility‐limited patients. They valued the scheduling flexibility, extended hours, and inclusion of family members remotely, alongside gaining insights into patients’ home environments for personalized care [43, 44, 50]. Efficiency gains like reduced wait times and minimized infection exposure were frequently cited, and clinicians acknowledged targeted solutions like providing low‐cost devices could bridge access gaps [40, 43, 50]. Conversely, negative sentiments revealed significant unresolved challenges exacerbating inequities. Technical failures, including unstable internet and poor audio quality, routinely disrupted care and lacked real‐time support [40, 43]. Socioeconomic, cognitive, physical, sensory, and language barriers further limited accessibility and degraded interaction quality compared to in‐person visits, while scheduling errors and privacy concerns undermined engagement [40, 43]. Neutral observations objectively noted telehealth magnifies preexisting disparities, with older adults, non‐English speakers, and socioeconomically disadvantaged groups consistently experiencing lower quality virtual care [43, 44, 50]. Rural patients were hindered by inadequate connectivity, and clinicians stressed that success depends on hybrid models and addressing root causes like broadband access and device affordability [43, 44, 50].

Nurses showed evenly distributed sentiment (33% each positive, negative, neutral). Negative sentiments highlighted critical infrastructure gaps impeding access, such as geographic barriers forcing long travel for specialized care and financial burdens from diagnostic costs, underscoring unmet needs for remote solutions [53]. Positive sentiments emphasized digital tools directly overcoming accessibility barriers, noting telemedicine effectively complemented infrequent in‐person visits with timely consultations and strong acceptance, while ePHRs streamlined information retrieval, reducing delays and enhancing efficiency [53, 54]. Neutral sentiments objectively outlined transitional benefits, noting ePHRs simplified accessing historical data compared to physical archives and remote prescription management reduced time burdens for rural patients, reflecting measured recognition of incremental progress rather than transformative change [44, 53].

Caregivers stated the patient strongly preferred face‐to‐face interactions due to significant hearing and eyesight impairments, as physical presence enabled reliance on lip‐reading and observing facial expressions for understanding, which digital alternatives could not support [49].

Renal technicians highlighted digital tools enabling faster problem resolution, often eliminating in‐person visits, specifically noting the benefit of visual communication where patients could show error messages or point to components, allowing remote diagnosis [55].

#### 3.4.2. Communication Dynamics

From Figure 4(b), patients expressed predominantly positive sentiment (64.8% positive, 21.6% negative, 13.6% neutral), valuing telemedicine’s convenience and flexibility for reducing stress and enabling comfortable home‐based consultations. Patients highlighted better communication through patient‐reported outcomes (PROs), which helped organize thoughts and prepare for discussions, while established rapport with physicians enhanced comfort and effectiveness during virtual visits [40, 42, 46, 56]. Many noted more focused conversations with less rushed providers and appreciated sustained contact for ongoing chronic condition management [40–44, 46–52, 54, 57–59]. However, negative sentiments revealed significant challenges, including perceived loss of personal connection due to absent nonverbal cues and physical presence, leading to mistrust during serious discussions [43, 44, 46, 50, 51, 59]. Patients also expressed frustration with untimely feedback after consultations or data submissions [42, 46, 48]. Concerns about depersonalization—feeling reduced to “just a number”—and data security emerged, though the latter elicited limited practical concern [40, 42, 43, 46, 48–50]. Neutral observations described factual experiences with digital tools, noting variable communication quality across platforms and providers while acknowledging both benefits and limitations without strong judgment [40–43, 46, 51, 53, 57, 58].

Clinicians showed moderately positive sentiment (42.4% positive, 33.3% negative, 24.2% neutral), recognizing digital tools could enhance communication through family involvement in telehealth visits, fostering collaborative care. Established clinician–patient relationships enabled effective follow‐ups, while remote monitoring deepened empathy by revealing patients’ daily dialysis experiences, promoting patient‐centered approaches [40, 43, 44]. Structured digital tools (ePROMs, ePHR) modernized information exchange, and clear responsibility definitions on platforms reduced miscommunication [40, 43]. Conversely, clinicians expressed significant concerns about eroded interpersonal connections, citing the loss of body language, eye contact, and environmental context as impairing authentic emotional assessment and risking superficial interactions or misdiagnosis [40, 43, 44, 50, 53, 56]. Technical issues like audio/video lag exacerbated these sensory deficits, while sensitive conversations (e.g., breaking bad news) became challenging without physical presence to provide comfort [40, 43, 44, 50, 53, 56]. Patients’ reluctance to disclose symptoms due to virtual power imbalances and critiques of impersonal tools—such as PROMs replacing dialogue with rigid questionnaires—further hindered communication [40, 43, 53, 56]. Neutral perspectives acknowledged pragmatic adaptations: addressing elderly patients’ skepticism through cultural sensitivity, managing environmental distractions inherent to remote settings, and developing workarounds like postvisit summaries via EMRs for clarity. While digital tools opened new avenues for difficult discussions, clinicians emphasized the irreplaceable value of therapeutic touch and noted privacy concerns often lacked justification, underscoring the need for hybrid models balancing efficiency with human connection [40, 43, 44, 50, 53].

Nurses demonstrated a strongly positive sentiment (80.0% positive, 20.0% negative, 0% neutral). Collectively, nurses articulated a journey from uncertainty to validated utility, where technology democratized communication by mitigating hierarchical gaps, building bidirectional trust, and creating space for both efficiency and empathy [54].

Caregivers noted that telephone‐based telehealth enabled their participation in appointments, reducing worry about the patient missing critical information when absent. Remote monitoring also helped avoid being perceived negatively (“telling tales”) by the patient when reporting health information [43, 59].

Providers emphasized the importance of maintaining established doctor–patient relationships, particularly for complex cases. They valued digital communication (e.g., phone calls) for offering flexible, immediate consultation (“pick your phone up and talk”) as an alternative to written exchanges when needed [45].

#### 3.4.3. Workflow Integration

From Figure 4(c), patients expressed moderately positive sentiment (53.5% positive, 32.6% negative, 14.0% neutral), appreciating telehealth’s convenience and flexibility in reducing travel burdens and saving time. They recognized telemonitoring’s potential for cost‐effective chronic disease management and valued the integration of tools like ePROMs during dialysis for simplifying communication and care management [40, 43, 46, 48, 49, 53, 54]. Trust in platforms developed by reputable institutions (e.g., government, hospitals) was emphasized as crucial for adoption [40, 46, 48, 49, 58, 59]. Conversely, negative sentiments highlighted significant barriers: frustration with technical issues, concerns about overburdening local doctors and slowing information flow, and disappointment when clinicians failed to utilize provided patient data [43, 45, 49, 51, 53, 56, 58–60]. Neutral perspectives noted reduced postpandemic telemedicine usage and varying comfort with communication modes (e.g., preferring phone calls), while offering practical suggestions like timed reminders and reliable message delivery to enhance intervention integration [46, 53, 54, 60].

Clinicians demonstrated predominantly positive sentiment (60.7% positive, 14.3% negative, 25.0% neutral), endorsing digital tools for enhancing clinical workflows through efficiency gains such as focused consultations, faster data tracking, and reduced communication burdens via automation [40, 53, 56]. Key implementation drivers included intuitive data visualization, flexible administration timing, and patient‐specific frequency [40, 50]. Stakeholder engagement—clinician buy‐in, cross‐disciplinary collaboration, and strong leadership support—was framed as foundational, with tools viewed as professional aids aligning with healthcare’s digital evolution [40, 50, 51]. Clear guidelines and simplified workflows with expert support were further facilitators [40]. However, negative sentiments revealed critical barriers: alert fatigue within overloaded EHR systems, concerns about increased workload and time constraints, and fears that tools could strain psychological resources and trigger unsustainable referral volumes [40, 53]. Neutral observations stressed contextual implementation needs, noting workflow adaptations (e.g., preconsultation completion), technical compatibility requiring role‐based access, standardized team understanding, and context‐dependent utility based on consultation styles, multidisciplinary integration, and disease applicability [40, 53, 56].

Nurses showed cautiously positive sentiment (53.8% positive, 15.4% negative, 30.8% neutral), emphasizing efficiency gains such as significant time savings through reduced call durations (75%) and automated education, freeing time for direct care [53, 55]. Workflow flexibility enabled multitasking via remote monitoring, while system familiarity with existing HIS platforms facilitated adoption [53, 55]. Transformative care model improvements generated strong optimism [54, 55]. Negative sentiments exposed systemic barriers: inadequate training implementation due to deprioritization or interruptions, and role ambiguity creating tension over responsibilities for complex decisions (e.g., drug discontinuation) [51, 53]. Neutral perspectives pragmatically addressed integration sustainability, noting that interoperability required seamless HIS–new platform data exchange despite logistical complexity, and stressing that inconsistent physician engagement limited tool utility. While remote monitoring enabled autonomous problem‐solving, a time‐efficiency paradox emerged: Nurses acknowledged long‐term benefits but cited immediate time constraints as adoption barriers [44, 53].

Providers stated digital tools offered legal documentation and decision‐making confidence, but expressed major concerns about workflow sustainability [45]. High volumes of digital queries and a lack of compensation were significant barriers [45]. They strongly opposed disconnected systems, citing duplicated data entry into separate platforms as “very labour intensive” and the “biggest barrier,” emphasizing the need for systems “easy to use” (“click‐click‐click”) and integrated with existing EMRs [45].

Renal Matron reported digital interventions reduced in‐person nursing time for initial home dialysis training from 3 days to one day [55].

Caregiver emphasized that digital tools (e.g., ePHRs) required physician authorization and support to gain patient trust [53].

#### 3.4.4. Patient Empowerment

From Figure 4(d), patients expressed strongly positive sentiment (64.6% positive, 11.5% negative, 24.0% neutral), highlighting digital tools’ role in enabling self‐management and enhancing agency through actionable knowledge. Simplified dietary guidelines (e.g., “red/yellow/green” food categorization) and quantifiable portion recommendations built confidence in daily decisions, while recurrent feedback via texts or apps reinforced health goals and motivated behavior changes [42, 47, 52, 53, 57, 58, 60, 61]. Patients particularly valued personalized features like tailored medication reminders, lab result access, and individualized recipes that fostered autonomy [57, 58, 61]. The emotional dimension was prominent: Remote interactions alleviated isolation, with messages perceived as proof “someone cares,” boosting self‐esteem and accountability. Timely digital support demystified complex rules for newly diagnosed or dialysis‐transitioning patients, providing clarity on “what I should be doing” [40, 42, 51–54, 57, 61, 62]. However, negative sentiments revealed empowerment barriers, including frustration with unreliable online information, causing conflicting advice and eroded trust [52, 58]. Technical limitations like standardized content felt irrelevant, leading patients to “discard” advice clashing with routines. Tools requiring disease‐specific knowledge (e.g., PRO questionnaires) caused anxiety about “how to respond” among those lacking foundational understanding, while rigid tools disrupted established coping strategies [42, 52, 57, 61]. Neutral perspectives reflected pragmatic acceptance, acknowledging convenience for tracking lab results or accessing nutritional data “at your fingertips,” but emphasizing that tools cannot replace human expertise [42, 43, 52, 53, 57, 58, 61, 62]. Patients noted digital records offered clarity (“written out forever”) yet stressed dependence on dietitian guidance for complex adjustments, viewing tools as useful transiently during dietary transitions before habits solidified [42, 43, 52, 53, 57, 58, 61, 62].

Clinicians demonstrated predominantly positive sentiment (58.8% positive, 11.8% negative, 29.4% neutral), emphasizing digital tools’ therapeutic value in fostering empowerment through psychological support and self‐management capabilities. Remote monitoring and ePHRs provided critical reassurance, creating a sense of continuous oversight and safety at home that alleviated anxiety [40, 43, 44, 50, 53]. Telehealth and symptom‐tracking systems enabled active patient participation—such as sharing vital signs or controlling data access—while literacy‐adapted educational content (e.g., videos for low eHealth literacy patients) reduced stress among early‐stage patients [40, 43, 44, 50]. Clinicians praised ePROMs for shifting care toward holistic quality‐of‐life metrics and reinforcing continuity, where prior documented conversations streamlined goal‐aligned discussions during health declines [40, 43, 44, 50]. Conversely, negative sentiments revealed apprehensions about unintended consequences: Fears that ePROMs might create unrealistic outcome expectations leading to dissatisfaction or mistrust, and concerns about medicalizing normal experiences (e.g., pathologizing natural stress through excessive psychological monitoring) [40, 44]. Neutral perspectives addressed implementation feasibility, acknowledging tools might encourage self‐reflection and extend postdischarge support but highlighting contextual barriers [40, 44, 53, 56]. Clinicians questioned the burden on severely ill patients struggling to engage with ePROMs, framing empowerment strategies as double‐edged—offering structural benefits while demanding careful calibration to individual capabilities [40, 44, 53, 56].

Nurses showed overwhelmingly positive sentiment (70.0% positive, 10.0% negative, 20.0% neutral), focusing on empowerment as a core benefit. They emphasized remote monitoring and real‐time data access fostered a deeper understanding of dialysis, fluid status, and health, transforming patient roles and promoting “shared care” [44, 51, 53, 56]. These tools provided critical reassurance to anxious patients transitioning to home‐based care, while tailored educational content for different CKD stages reduced provider burden and increased patient efficacy [44, 51, 53, 56]. Nurses celebrated patient‐driven features like messaging systems as tools that “give power to patients,” directly linking technology to enhanced autonomy and framing it as adaptable to individual capacities despite variable readiness [44, 51, 53, 56]. The single negative sentiment centered on privacy control as a non‐negotiable prerequisite, arguing that without exclusive patient authority over ePHR access, fear of unauthorized sharing could cause information withholding—fundamentally undermining trust and agency [53]. Neutral perspectives pragmatically addressed structural limitations, noting low eHealth literacy prevented engagement with complex systems, and physical barriers like visual impairment necessitated adaptive solutions (e.g., tablet zoom functions), underscoring that empowerment initiatives must accommodate varying capabilities to be inclusive [53, 56].

Renal services manager stated digital interventions allowed patients to maintain normal daily activities (“carrying his life on as normal”) by eliminating frequent, lengthy travel [55].

Consultant noted digital tools encouraged patients to actively reflect on their health status (“think about how they are doing”) and engage with multiple aspects of their condition (“the different aspects they ask about”) [51].

#### 3.4.5. Clinical Effectiveness

From Figure 4(e), patients expressed moderately positive sentiment (54.3% positive, 26.1% negative, 19.6% neutral), valuing digital interventions for convenience, accessibility, and psychological reassurance. They highlighted time‐saving benefits in remote communication for prescription refills and minor issues, appreciating telemedicine’s role in bridging geographical barriers, and providing timely advice [40, 41, 43, 46, 53, 54]. Telemonitoring fostered confidence through regular professional check‐ins, while ePROM systems were favored for early detection of health deterioration and psychological distress [40, 42, 46, 48, 54, 58, 62]. Patients reported satisfaction with telehealth’s adequacy during stable health periods, noting virtual formats could be as effective as in‐person visits for routine dialysis discussions or lab follow‐ups [40, 42, 46, 48, 54, 58, 62]. However, negative sentiments emphasized significant diagnostic limitations: Remote assessments were perceived as inadequate for conditions requiring visual or hands‐on evaluation (e.g., leg swelling, skin changes), with concerns about compromised diagnostic accuracy due to inferior equipment or absent face‐to‐face interaction [48, 59]. Patients preferred in‐person care for condition changes, deterioration, or treatment decisions like dialysis initiation, citing challenges in self‐performing routine assessments (e.g., fluid retention monitoring) [49, 59]. Variable care quality and reduced disease education/reassurance compared to in‐person interactions were additional concerns [42, 43, 51, 53]. Neutral perspectives acknowledged context‐dependent utility, recognizing telehealth’s effectiveness for stable health or minor issues but stressing its unsuitability for complex concerns [41, 46, 49]. Patients advocated hybrid models combining virtual and physical care, noting that effectiveness varies by technological proficiency, equipment access, and platform features [42, 46, 49, 59].

Clinicians demonstrated predominantly negative sentiment (50.0% negative, 36.4% positive, 13.6% neutral), expressing fundamental concerns about diagnostic rigor. Positive acknowledgments focused on digital tools’ potential to enhance care quality through evidence‐based adoption (e.g., ePROM impact validation before implementation) and research engagement [40, 53]. Clinicians highlighted benefits like systematic symptom detection for earlier issue identification, adjunctive clinical value complementing traditional care, and symptom‐focused applications in palliative settings [40, 53]. Tools enabling care coordination through centralized data access were seen as efficiency‐boosting, though optimism was tempered by treatment trade‐offs between clinical targets and patient well‐being, alongside critical emphasis on data accuracy to mitigate risks from incorrect patient‐entered information [40, 53]. Negative sentiments centered on compromised diagnostic rigor and safety: Diagnostic limitations and uncertainty arose from the inability to perform physical exams or obtain vital signs via telehealth, directly linking to care quality compromises [40, 43, 44, 50]. Data interpretation challenges and clinical relevance ambiguity created unease about translating PROMs into actionable insights [40, 43]. Significant apprehension included litigation risks if digital data remained unaddressed, prognostic uncertainty hindering goal‐of‐care discussions, resource efficiency doubts given persistent lab needs, and limited clinical utility of PROMs for replacing hard risk factors in stratification [40, 43, 44, 50]. Neutral sentiment points out the clinical ambiguity due to underlying safety concerns, although remote monitoring aided adherence tracking [40, 44].

Nurses showed a strongly positive sentiment (75.0% positive, 12.5% negative, 12.5% neutral), emphasizing workflow transformation benefits. Real‐time visual assessment capabilities (e.g., inspecting cloudy dialysis bags via video) enabled immediate troubleshooting during critical moments, reducing readmissions, and enabling “quicker problem resolution” [53, 57]. Centralized patient data access eliminated redundant tests and paper inefficiencies, shortening hospital stays and lowering costs [53, 57]. This integration shifted care from reactive to proactive; remote monitoring allowed earlier issue detection and preemptive treatment adjustments, hailed as “singularly the most effective IT innovation” for efficiency gains [44, 53, 57]. However, one negative sentiment highlighted data reliability concerns, questioning whether on‐screen information during consultations was “accurate and applicable” and warning that flawed data could compromise problem‐solving [44]. A neutral perspective pragmatically addressed data timeliness, noting that delayed symptom surveys diminished clinicians’ ability to act meaningfully [56]. Collectively, nurses framed digital tools as potent enablers of clinical effectiveness through accelerated decisions and preventive care, but stressed gains depend on real‐time, accurate data—effectiveness diminishes when information currency lags behind clinical needs [44, 53, 55, 56].

Caregiver noted certain tests (e.g., blood work, CAT scans) cannot be performed remotely but reported remote monitoring made patients feel “more relaxed and supported” through constant clinical oversight [43, 59].

Renal technical services manager described telehealth enabling a nurse to remotely guide a patient through a needle dislodgement emergency—calming the patient, clamping the line, disconnecting dialysis, and stopping the machine—thereby preventing hospitalization [55].

Consultant stated digital tools allowed better preparation for phone consultations but noted the inability to visually assess patient well‐being compared to in‐person meetings [51].

### 3.5. Results of Selection of ERIC Strategies

The qualitative systematic review mapped barriers and facilitators of DHIs for CKD management to the CFIR constructs, such as intervention characteristics, inner setting, characteristics of individuals, and process. These mappings informed the selection of strategies from the ERIC tool to address identified barriers. The selection process was iterative, involving team consensus and validation by experts in CKD, digital health, and implementation science, with an audit trail ensuring transparency and rigor.

**FIGURE 5:**
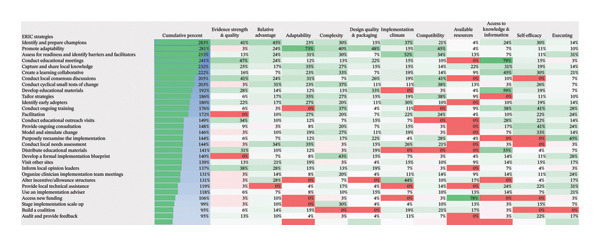
Top 30 of the ERIC strategies.

Figure 3 presents the top 30 ERIC strategies for implementing DHIs in CKD care, their cumulative priority scores, and scores across key CFIR‐aligned dimensions (the full edition could be found in Supporting file 4). The first column lists the strategies, while the second column, “cumulative percent,” reflects the overall priority based on the sum of scores across dimensions. “Evidence strength and quality” indicates the robustness of supporting evidence. “Relative advantage” measures perceived benefits over existing practices. “Complexity” assesses implementation difficulty. “Implementation climate” evaluates organizational readiness and support. “Compatibility” reflects alignment with existing workflows. “Executing” measures the feasibility of strategy execution (Figure 5).

For example, “identify and prepare champions” achieved the highest cumulative score of 283% (which represents a 25.7% overall proportional agreement out of the theoretical maximum of 1100%), underscoring its priority. This strategy benefits from strong evidence strength and quality (0.41), high relative advantage (0.45), and moderate complexity (0.30), alongside strong compatibility (0.21) and execution feasibility (0.14). This indicates that leveraging influential champions is both evidence‐based and practical for driving adoption. Similarly, “promote adaptability” (281%) scored highly due to its adaptability (0.73) and low complexity (0.40), making it suitable for tailoring interventions to diverse CKD care settings.

The top 10 strategies are as follows:

1. Identify and prepare champions (cumulative: 283%; proportion: 25.7%): engaging leaders to advocate for DHIs, supported by strong evidence and compatibility.
2. Promote adaptability (cumulative: 281%; proportion: 25.5%): customizing interventions to fit local needs, with high adaptability and moderate complexity.
3. Assess for readiness and identify barriers and facilitators (cumulative: 253%; proportion: 23.0%): Evaluating organizational readiness, with strong implementation climate support.
4. Conduct educational meetings (cumulative: 241%; proportion: 21.9%): delivering training to enhance stakeholder knowledge, with high self‐efficacy impact.
5. Capture and share local knowledge (cumulative: 232%; proportion: 21.1%): utilizing local insights, with balanced scores across dimensions like access to knowledge.
6. Create a learning collaborative (cumulative: 222%; proportion: 20.2%): fostering stakeholder networks, supported by strong execution feasibility.
7. Conduct local consensus discussions (cumulative: 205%; proportion: 18.6%): building agreement, with notable compatibility and evidence strength.
8. Conduct cyclical small tests of change (cumulative: 203%; proportion: 18.5%): piloting interventions iteratively, with moderate complexity and compatibility.
9. Develop educational materials (cumulative: 192%; proportion: 17.5%): creating resources to support engagement, with strong self‐efficacy.
10. Tailor strategies (cumulative: 186%; proportion: 16.9%): customizing approaches to barriers, with high compatibility and adaptability.

## 4. Discussion

This systematic review represents the first comprehensive integration of computational sentiment analysis with qualitative synthesis to decode the affective dimensions of stakeholder experiences regarding DHIs in CKD management. By analyzing 23 qualitative studies across diverse healthcare settings, we quantified sentiment polarity across five critical domains and mapped these insights into evidence‐based implementation strategies. Our findings reveal profound tensions between stakeholder groups, expose domain‐specific adoption barriers, and provide a roadmap for optimizing DHI implementation through the ERIC framework.

### 4.1. Key Findings and Interpretation

#### 4.1.1. Sentiment Polarity Reveals Critical Stakeholder Tensions

Sentiment analysis uncovered profound tensions between stakeholder groups, highlighting divergent priorities and barriers to DHI adoption in CKD management. Patients expressed strong enthusiasm for patient empowerment (64.6% positive sentiment), valuing self‐management tools like personalized medication reminders and dietary apps that fostered autonomy, while communication dynamics elicited predominantly positive sentiment (64.8% positive) due to telemedicine’s convenience and perceived focus during virtual consultations. In stark contrast, clinicians exhibited significant skepticism toward clinical effectiveness, with 50.0% negative sentiment driven by concerns about diagnostic limitations (e.g., inability to perform physical exams) and ambiguity in translating patient‐reported data into actionable insights. This fundamental tension—between patients’ prioritization of convenience/agency and clinicians’ emphasis on diagnostic rigor—threatens the perceived legitimacy of DHIs in clinical decision‐making.

Accessibility inequities further exposed systemic fault lines. While clinicians overall reported positive sentiment on accessibility (58% positive), their negative (23%) and neutral (19%) comments consistently highlighted how DHIs risked exacerbating disparities: rural patients faced connectivity gaps, older adults struggled with technical interfaces, and socioeconomically disadvantaged groups experienced lower quality virtual care. This aligned with patients’ accessibility frustrations (35% negative sentiment), where socioeconomic barriers (e.g., hidden costs, device affordability) eroded trust. Nurses, despite cautiously positive views on workflow integration (53.8% positive), voiced critical concerns about role ambiguity and inadequate training (15.4% negative), revealing tensions between efficiency gains and organizational readiness.

Marginalized perspectives amplified these tensions. Caregivers emphasized sensory limitations (e.g., hearing/vision impairments) that made patients dependent on in‐person cues like lip‐reading—a need unmet by current DHIs. Renal technicians, conversely, highlighted the irreplaceable value of visual communication (e.g., remotely diagnosing device errors via video), underscoring how technical roles prioritize pragmatic problem‐solving over theoretical workflows.

Policymakers should reform reimbursement structures to compensate digital consultations and fund broadband infrastructure, countering socioeconomic exclusion identified in negative sentiments across groups [63, 64]. Crucially, our findings both corroborate and significantly advance the current evidence base regarding DHI implementation. Consistent with umbrella reviews of chronic disease management by Taylor et al. [27] and Jeddi et al. [24], we identified workflow disruption and clinician skepticism as primary implementation barriers. However, our sentiment analysis extends this literature by uniquely quantifying the profound affective depth of this resistance—revealing that 50% of clinician sentiment regarding clinical effectiveness is overtly negative, driven primarily by diagnostic anxiety rather than mere technical friction. Conversely, our findings on patient empowerment strongly align with Lightfoot et al.’s [22, 23] work on increased patient activation in CKD, yet we add a critical caveat: this empowerment is highly fragile. Our data demonstrate that positive sentiments toward digital autonomy are rapidly eroded by negative affective experiences linked to digital exclusion and systemic accessibility barriers—a nuanced emotional trajectory often obscured in purely thematic syntheses. Developers must prioritize interoperability with existing EHRs to eliminate duplicative data entry (cited as the “biggest barrier” by providers) and integrate adaptive interfaces (e.g., zoom functions for visually impaired users) to mitigate accessibility inequities [65, 66]. Policymakers should reform reimbursement structures to compensate digital consultations and fund broadband infrastructure, countering socioeconomic exclusion identified in negative sentiments across groups [67–69].

#### 4.1.2. ERIC Strategies Address Domain‐Specific Barriers

Theoretically, the stakeholder tensions identified in our review expose critical misalignments across core CFIR domains. We observed that rigid intervention characteristics (e.g., standardized ePROMs, complex user interfaces) frequently clash with the inner setting (e.g., strained clinician workflows, alert fatigue) and the characteristics of individuals (e.g., varying patient digital literacy, caregivers’ sensory limitations). By using CFIR to diagnose these theoretical disconnects, our prioritization of ERIC strategies moves beyond generic recommendations to offer targeted, theoretically grounded interventions. Our ERIC strategy prioritization directly responds to sentiment‐derived challenges:

Top‐ranked strategies (e.g., identify and prepare champions 283%, promote adaptability 281%) target CFIR constructs like implementation climate and compatibility.

For example, theoretically, “promote adaptability” functions as a bridging strategy to harmonize intervention characteristics with diverse outer setting patient contexts (e.g., adapting interfaces for visually impaired users). Similarly, “identify and prepare champions” serves as a targeted intervention to modify the implementation climate within the inner setting, directly neutralizing the negative clinician affect we quantified by utilizing peer influence to demonstrate evidence‐based utility [70, 71].

Workflow integration barriers (e.g., clinician alert fatigue, role ambiguity) align with strategies like developing educational materials and conducting local consensus discussions to standardize protocols and clarify responsibilities. Accessibility inequities necessitate tailored strategies, such as providing subsidized devices or hybrid care models for marginalized groups—directly addressing negative sentiments about exclusion.

Notably, the high cumulative scores for assess for readiness (253%) and capture local knowledge (232%) underscore the need for preimplementation stakeholder engagement to surface latent concerns (e.g., clinician fears about litigation).

### 4.2. Methodological and Practical Implications

#### 4.2.1. Advancing Implementation Science Methodology

Our dual‐method approach demonstrates that sentiment analysis adds critical value beyond traditional thematic synthesis by:

1. Quantifying emotional undertones that influence behavior (e.g., neutral patient attitudes toward accessibility reflected pragmatic resignation rather than engagement).
2. Exposing disparities in stakeholder priorities (e.g., clinicians’ workflow concerns vs. technicians’ focus on visual problem‐solving).
3. Identifying hidden risks, such as “pragmatic acceptance” masking latent dissatisfaction.

This methodology is transferable to other chronic conditions (e.g., diabetes, heart failure) where stakeholder sentiment heterogeneity impacts DHI adoption. Future studies should validate sentiment algorithms against in‐depth interviews to capture context‐specific nuances like sarcasm or cultural expressions of emotion.

#### 4.2.2. Actionable Recommendations for Stakeholders

The ERIC strategies we prioritized—led by identify and prepare champions (cumulative score: 283%) and promote adaptability (281%)—provide actionable pathways to address sentiment‐identified barriers. Critically, these strategies directly target affective pain points revealed by our analysis:

Clinicians’ negative sentiments on clinical effectiveness (−50.0%, driven by diagnostic uncertainty) necessitate developing educational materials (ERIC #9) to validate tool accuracy and conducting educational meetings (ERIC #4) demonstrating evidence‐based utility.

Patients’ accessibility frustrations (35% negative sentiment on tech barriers) demand tailored strategies (ERIC #10) to socioeconomic contexts—e.g., combining providing ongoing consultation with low‐cost device provisioning to alleviate distrust from “hidden costs.”

Nurses’ cautious workflow positivity (53.8% positive) is amplified by capture and share local knowledge (ERIC #5), transforming their efficiency gains into implementation momentum.

Stark sentiment misalignments (e.g., patients’ +64.6% empowerment enthusiasm vs. clinicians’ workflow apprehensions) are bridged through creating a learning collaborative (ERIC #6), fostering empathy by sharing emotional narratives across stakeholder groups.

To optimize strategy selection, implementers should:

1. Employ preference elicitation methods like discrete choice experiments or best‐worst scaling to quantify stakeholder trade‐offs (e.g., weighing workflow disruption against long‐term efficiency).
2. Conduct context‐specific barrier analyses using CFIR‐sentiment mappings to target high‐impact domains (e.g., focusing on accessibility in regions with connectivity gaps).
3. Iteratively refine strategies through cyclical small tests of change (ERIC #8), particularly for interventions triggering negative sentiments (e.g., piloting ePROMs with clinician champions to mitigate workflow concerns).

### 4.3. Perspectives for Clinical and Assistive Practice

Building upon the multidimensional and multidisciplinary framework introduced earlier, our findings offer highly concrete perspectives for modern clinical and assistive practice. The stark sentiment tensions we identified—particularly the high enthusiasm for patient empowerment (+64.6%) contrasted with clinician skepticism regarding remote diagnostic effectiveness (−50.0%)—underscore that DHIs cannot function as standalone technical solutions; they must be organically embedded into existing care pathways.

First, regarding the competencies of nephrology nurses, our data position them as the crucial nexus of DHI integration. While nurses reported workflow efficiencies (+53.8%), they also highlighted role ambiguity. In practice, this means health systems must explicitly redefine nursing competencies to include digital care coordination. By applying our top‐ranked ERIC strategy, *Identify and prepare champions*, advanced practice nephrology nurses can be empowered to lead multidisciplinary teams, triage remote monitoring alerts, and bridge the communication gap between patients and skeptical physicians.

Second, within dedicated care programs for patients starting dialysis, DHIs offer unprecedented value. The profound patient anxiety during this transition can be mitigated by the continuous reassurance of remote symptom tracking and ePROMs. However, as our sentiment analysis revealed, this empowerment is fragile and easily disrupted by technological barriers. Therefore, applying the *Promote adaptability* strategy is essential in assistive practice—ensuring that digital educational materials and fluid–monitoring interfaces are highly tailored to the specific cognitive and visual needs of patients with ESRD.

Finally, in the context of ongoing frailty assessments, the deployment of targeted DHIs facilitates proactive, rather than reactive, multidisciplinary care. Continuous digital data streams allow dietitians, social workers, and nephrologists to detect early signs of functional decline before scheduled in‐person visits. To overcome the diagnostic anxieties voiced by clinicians in our study, clinical practice must pivot toward hybrid care models. In these models, DHIs handle routine psychosocial reassurance and data collection, reserving face‐to‐face consultations for complex physical examinations and critical treatment decisions. Ultimately, aligning these technological tools with the psychosocial realities of stakeholders is what transforms digital health from an administrative burden into a potent enabler of modern, equitable CKD care.

### 4.4. Strengths and Limitations

Strengths include rigorous PROSPERO‐registered methods, stakeholder‐stratified sentiment quantification, and ERIC–CFIR mapping validated by CKD/digital health experts. Limitations encompass the following: exclusion of non‐English studies and gray literature; nuanced sentiment (e.g., sarcasm) potentially misclassified by lexicon‐based VADER; and underrepresentation of low‐resource settings in included studies. Furthermore, we acknowledge a limitation regarding our implementation mapping: The standard 73 ERIC strategies sometimes lacked the specificity required to address unique digital health barriers (e.g., software interoperability, digital interface design, or specific digital literacy gaps). Because we strictly adhered to the established CFIR–ERIC matching tool to maintain methodological rigor, we did not formulate novel eHealth strategies to fill these gaps. Future research should prioritize adapting or expanding the ERIC taxonomy specifically for DHIs to provide more granular, tech‐specific implementation guidance.

## 5. Conclusion

This review demonstrates that stakeholder sentiments—rather than mere technical functionality—are the primary determinants of DHI success within modern, multidisciplinary CKD care. To effectively support critical clinical practices such as routine frailty assessments and dedicated dialysis transition programs, healthcare systems must reconcile stark stakeholder tensions. Clinician skepticism regarding remote diagnostic validity, patient fears of depersonalization, and the urgent need to redefine nephrology nursing competencies demand targeted, multifaceted solutions. Our prioritized ERIC strategies offer a robust implementation blueprint, emphasizing clinical champion engagement, workflow adaptability, and equity‐driven design.

However, these findings and their inherent limitations must be framed within the broader international academic debate on digital health equity and global healthcare delivery. While our identified strategies are robust, their universal applicability remains constrained by profound international variations. The global digital divide, disparities in transnational broadband infrastructure, and contrasting national healthcare reimbursement models heavily influence the feasibility of DHI adoption. Furthermore, cross‐national differences in the scope of nursing practice mean that relying on nurses as “digital champions” may face varying structural barriers across different global health systems.

Consequently, future research must move beyond localized contexts to include cross‐cultural, international real‐world trials that adapt these implementation strategies to diverse socioeconomic and legislative environments. By acknowledging these global complexities and systematically addressing both affective and practical barriers, international healthcare systems can successfully harness DHIs to transform CKD from a trajectory of inevitable decline into an era of proactive, empowered, and equitable multidisciplinary management.

## Author Contributions

Xutong Zheng: conceptualization; data curation; formal analysis; methodology; project administration; software; supervision; validation; visualization; roles/writing–original draft.

Liuxuan Xie: project administration; resources; software; supervision.

Yingshuo Liu: screening and extracting the document.

Qiuying Yu: screening and extracting the document.

Han Zhang: screening and extracting the document.

Xiao Zhang and Qiuying Yu: screening and extracting the document.

Aiping Wang: conceptualization, and review and editing.

## Funding

No funding was received for this research.

## Ethics Statement

The authors have nothing to report.

## Consent

The authors have nothing to report.

## Conflicts of Interest

The authors declare no conflicts of interest.

## Supporting Information

Additional supporting information can be found online in the Supporting Information section.

## Supporting information


**Supporting Information 1** Supporting Information 1: Details of the search strategies for each database.

**Supporting Information 2** Supporting Information 2: Details of the quality appraisal results.

**Supporting Information 3** Supporting Information 3: Details of CERQual Evidence Profile.

**Supporting Information 4** Supporting Information 4: Details of CFIR–ERIC strategies.

## Data Availability

Research data will be shared with reasonable requests from the corresponding author, Aiping Wang.
