# Supplementary material for: When Tech Meets Touch: Multistakeholder Perspectives and Implementation Strategies for eHealth in Chronic Kidney Disease: A Systematic Review Using Computational Linguistics
Source: J Nurs Manag. 2026 May 13;2026:7612011. doi: 10.1155/jonm/7612011 (PMC13172292; doi:10.1155/jonm/7612011)
Supplement: Supplementary file 1 — Supporting Information 1 Supporting Information 1: Details of the search strategies for each database. [file JONM-2026-7612011-s001.docx]

## Supplemental file 1 Searching strategy

## Pubmed

| Search number | Query | Results |
| --- | --- | --- |
| 1 | "Kidney Failure, Chronic"[Mesh] | 104,391 |
| 2 | ((((((End-Stage Kidney[Title/Abstract]) OR (End Stage Kidney[Title/Abstract])) OR (Chronic Kidney Failure[Title/Abstract])) OR (End-Stage Renal[Title/Abstract])) OR (End Stage Renal[Title/Abstract])) OR (Chronic Renal Failure[Title/Abstract])) OR (ESRD[Title/Abstract]) | 86,036 |
| 3 | "Renal Insufficiency, Chronic"[Mesh] | 143,899 |
| 4 | (Chronic Kidney Insufficienc*[Title/Abstract]) OR (Chronic Renal Insufficienc*[Title/Abstract]) | 5,912 |
| 5 | "Renal Dialysis"[Mesh] | 131,598 |
| 6 | ((Dialyses[Title/Abstract]) OR (Hemodialys*[Title/Abstract])) OR (Dialysis[Title/Abstract]) | 179,112 |
| 7 | "Hemodialysis, Home"[Mesh] | 2,243 |
| 8 | "Peritoneal Dialysis"[Mesh] | 29,186 |
| 9 | "Peritoneal Dialysis, Continuous Ambulatory"[Mesh] | 10,197 |
| 10 | (CAPD[Title/Abstract]) OR (Continuous Ambulatory Peritoneal Dialysis[Title/Abstract]) | 9,273 |
| 11 | "Hemodialysis Units, Hospital"[Mesh] | 1,492 |
| 12 | #1 OR #2 OR #3 OR #4 OR #5 OR #6 OR #7 OR #8 OR #9 OR #10 OR #11 | 310,759 |
| 13 | cell phones[MeSH Major Topic] | 17,480 |
| 14 | Computers, Handheld[MeSH Major Topic] | 9,096 |
| 15 | internet[MeSH Major Topic] | 59,464 |
| 16 | Electronic Mail[MeSH Major Topic] | 1,515 |
| 17 | Telemedicine[MeSH Major Topic] | 44,075 |
| 18 | ((((((((((((((((((((((((((((Cell Phone*[Title/Abstract]) OR (Computer-Assisted[Title/Abstract])) OR (Computerized Health Record*[Title/Abstract])) OR (Electronic Health Record*[Title/Abstract])) OR (electronic information system[Title/Abstract])) OR (Health Communication[Title/Abstract])) OR (Health Informatic*[Title/Abstract])) OR (Information Technology[Title/Abstract])) OR (Information Technology Personnel[Title/Abstract])) OR (interactive digital game[Title/Abstract])) OR (Internet[Title/Abstract])) OR (Mobile health[Title/Abstract])) OR (Occupational Health Service*[Title/Abstract])) OR (online[Title/Abstract])) OR (on-line[Title/Abstract])) OR (personal digital assistant[Title/Abstract])) OR (Public Health Informatic*[Title/Abstract])) OR (Remote Consultation*[Title/Abstract])) OR (remote monitor*[Title/Abstract])) OR (Smartphone[Title/Abstract])) OR (Telehealth[Title/Abstract])) OR (Tele-health[Title/Abstract])) OR (Telemedicine[Title/Abstract])) OR (Tele-medicine[Title/Abstract])) OR (Telenursing[Title/Abstract])) OR (Tele-nursing[Title/Abstract])) OR (web based[Title/Abstract])) OR (webbased[Title/Abstract])) OR (World Wide Web[Title/Abstract]) | 567,636 |
| 19 | #13 OR #14 OR #15 OR #16 OR #17 OR #18 | 612,129 |
| 20 | (((("qualitative research"[MeSH Terms] ) OR (qualitative[Title/Abstract])) OR (experience[Title/Abstract])) OR (perspective[Title/Abstract])) OR (perception[Title/Abstract]) | 1,770,826 |
| 21 | ("Interviews as Topic"[Mesh]) OR (interview*[Title/Abstract]) | 531,559 |
| 22 | ("Focus Groups"[Mesh]) OR (focus group*[Title/Abstract]) | 80,925 |
| 23 | ("Grounded Theory"[Mesh]) OR (Grounded Theory[Title/Abstract]) | 17,302 |
| 24 | (Phenomenol*[Title/Abstract]) OR (content analysis[Title/Abstract]) | 90,902 |
| 25 | ("Anthropology, Cultural"[Mesh]) OR (Ethnograph*[Title/Abstract]) | 198,579 |
| 26 | #20 OR #21 OR #22 OR #23 OR #24 OR #25 | 2,287,098 |
| 27 | #12 AND #19 AND #26 | 645 |

## Embase

| No. | Query | Results |
| --- | --- | --- |
| 1 | 'kidney failure'/exp OR 'kidney failure':ab,ti | 617172 |
| 2 | 'kidney insufficiency':ti,ab OR 'maternal kidney failure':ti,ab OR 'renal failure':ti,ab OR 'renal insufficiency':ti,ab OR 'terminal kidney failure':ti,ab OR 'kidney failure':ti,ab OR 'chronic kidney disease':ti,ab OR 'end stage renal':ti,ab OR 'end stage kidney':ti,ab | 375997 |
| 3 | 'dialysis'/exp | 259126 |
| 4 | 'dialysis':ti,ab OR 'hemodialysis':ti,ab OR 'peritoneal dialysis':ti,ab | 268617 |
| 5 | 'telenursing'/exp OR 'medical informatics'/exp OR 'information technology'/exp OR 'occupational health service'/exp OR 'internet'/exp OR 'mobile phone'/exp OR 'online system'/exp | 262093 |
| 6 | 'interactive digital game':ab,ti OR 'electronic information system':ab,ti OR 'computer-assisted':ab,ti OR 'computerized health record':ab,ti OR 'telemedicine':ab,ti OR 'tele medicine':ab,ti OR 'telehealth':ab,ti OR 'tele health':ab,ti OR 'mobile health':ab,ti OR 'remote consultation':ab,ti OR 'electronic health records':ab,ti OR 'personal digital assistant':ab,ti OR 'public health informatics':ab,ti OR 'remote monitoring':ab,ti OR 'remote monitor':ab,ti OR 'on line':ab,ti OR 'web based':ab,ti OR 'smartphone':ab,ti OR 'cell phones':ab,ti OR 'online':ab,ti OR 'information technology personnel':ab,ti OR 'occupational health services':ab,ti OR 'internet':ab,ti OR 'world wide web':ab,ti OR 'health communication':ab,ti OR 'telenursing':ab,ti OR 'tele nursing':ab,ti OR 'health informatics':ab,ti OR 'information technology':ab,ti | 687515 |
| 7 | 'qualitative analysis'/exp OR (('analys*' NEAR/5 'qualitative'):ti,ab) OR 'experience'/exp OR 'experience':ti,ab OR 'perception'/exp OR 'perception':ti,ab OR 'interview'/exp OR 'interview*':ti,ab OR 'grounded theory'/exp OR 'grounded theory':ti,ab OR 'phenomenology'/exp OR 'phenomenolog*':ti,ab OR 'content analysis':ti,ab OR 'ethnography'/exp OR 'ethnogeography':ti,ab OR 'ethnography':ti,ab | 2819296 |
| 8 | #1 OR #2 OR #3 OR #4 | 851551 |
| 9 | #5 OR #6 | 800798 |
| 10 | #7 AND #8 AND #9 | 1687 |

## Web of science

| # | Search Query | Results |
| --- | --- | --- |
| 1 | ((((((((((((((((((((TS=(End-Stage Kidney)) OR TS=(Kidney Failure, Chronic)) OR TS=(End Stage Kidney)) OR TS=(Chronic Kidney Failure)) OR TS=(End-Stage Renal)) OR TS=(End Stage Renal)) OR TS=(Chronic Renal Failure)) OR TS=(ESRD)) OR TS=(Renal Insufficiency, Chronic)) OR TS=(Chronic Kidney Insufficienc*)) OR TS=(Chronic Renal Insufficienc*)) OR TS=(Renal Dialysis)) OR TS=(Dialyses)) OR TS=(Hemodialys*)) OR TS=(Dialysis)) OR TS=(Hemodialysis, Home)) OR TS=(Peritoneal Dialysis)) OR TS=(Peritoneal Dialysis, Continuous Ambulatory)) OR TS=(CAPD)) OR TS=(Continuous Ambulatory Peritoneal Dialysis)) OR TS=(Hemodialysis Units, Hospital) and Preprint Citation Index (Exclude – Database) | 554249 |
| 2 | ((((((((((((((((((((((((((((TS=(Cell Phone*)) OR TS=(Computer-Assisted)) OR TS=(Computerized Health Record*)) OR TS=(Electronic Health Record*)) OR TS=(electronic information system)) OR TS=(Health Communication)) OR TS=(Health Informatic*)) OR TS=(Information Technology)) OR TS=(Information Technology Personnel)) OR TS=(interactive digital game)) OR TS=(Internet)) OR TS=(Mobile health)) OR TS=(online)) OR TS=(on-line)) OR TS=(personal digital assistant)) OR TS=(Public Health Informatic*)) OR TS=(Remote Consultation*)) OR TS=(remote monitor*)) OR TS=(Smartphone)) OR TS=(Telehealth)) OR TS=(Tele-health)) OR TS=(Telemedicine)) OR TS=(Tele-medicine)) OR TS=(Telenursing)) OR TS=(Tele-nursing)) OR TS=(web based)) OR TS=(webbased)) OR TS=(World Wide Web)) NOT (SILOID==("PPRN")) | 3435563 |
| 3 | ((((((((((((((TS=(qualitative research)) OR TS=(qualitative)) OR TS=(experience)) OR TS=(perspective)) OR TS=(perception)) OR TS=(Interviews as Topic)) OR TS=(interview*)) OR TS=(Focus Groups)) OR TS=(focus group*)) OR TS=(Grounded Theory)) OR TS=(Grounded Theory)) OR TS=(Phenomenol*)) OR TS=(content analysis)) OR TS=(Anthropology, Cultural)) OR TS=(Ethnograph*) and Preprint Citation Index (Exclude – Database) | 9450093 |
| 4 | #1 AND #2 AND #3 | 2865 |

## EBSCO

1 SU End-Stage Kidney OR SU End-Stage Renal OR SU End-Stage Renal OR SU Chronic Renal Failure OR SU hemodialys* OR SU dialysis OR SU Peritoneal Dialysis SU Chronic Kidney Insufficienc* OR SU Chronic Renal Insufficienc*--44215

2 SU Cell Phone* OR SU Computer-Assisted OR SU Computerized Health Record* OR SU Electronic Health Record* OR SU electronic information system OR SU Health Communication OR SU Health Informatic* OR SU Information Technology OR SU Information Technology Personnel OR SU interactive digital game OR SU Internet OR SU Mobile health OR SU Occupational Health Service* OR SU online OR SU on-line OR SU personal digital assistant OR SU Public Health Informatic* OR SU Remote Consultation* OR SU remote monitor* OR SU Smartphone OR SU Telehealth OR SU Tele-health OR SU Telemedicine OR SU Tele-medicine OR SU Telenursing OR SU Tele-nursing OR SU web based OR SU webbased OR SU World Wide Web--497675

3.SU qualitative OR SU experience OR SU perspective OR SU perception OR SU Interview* OR SU Focus Group* OR SU Grounded Theory OR SU Phenomenol* OR SU content analysis OR SU Anthropology N3 Cultural OR SU Ethnograph*--1187133

4.AND—69

## SCOPUS

1. TITLE-ABS-KEY ( end-stage AND kidney ) OR TITLE-ABS-KEY ( kidney AND failure, AND chronic ) OR TITLE-ABS-KEY ( end AND stage AND kidney ) OR TITLE-ABS-KEY ( chronic AND kidney AND failure ) OR TITLE-ABS-KEY ( end-stage AND renal ) OR TITLE-ABS-KEY ( end AND stage AND renal ) OR TITLE-ABS-KEY ( chronic AND renal AND failure ) OR TITLE-ABS-KEY ( esrd ) OR TITLE-ABS-KEY ( renal AND insufficiency, AND chronic ) OR TITLE-ABS-KEY ( chronic AND kidney AND insufficienc* ) OR TITLE-ABS-KEY ( chronic AND renal AND insufficienc* ) OR TITLE-ABS-KEY ( renal AND dialysis ) OR TITLE-ABS-KEY ( dialyses ) OR TITLE-ABS-KEY ( hemodialys* ) OR TITLE-ABS-KEY ( dialysis ) OR TITLE-ABS-KEY ( hemodialysis AND home ) OR TITLE-ABS-KEY ( peritoneal AND dialysis ) OR TITLE-ABS-KEY ( peritoneal AND dialysis AND continuous AND ambulatory ) OR TITLE-ABS-KEY ( capd ) OR TITLE-ABS-KEY ( continuous AND ambulatory AND peritoneal AND dialysis ) OR TITLE-ABS-KEY ( hemodialysis AND units, AND hospital) -- 515,789
2. TITLE-ABS-KEY (Cell Phone*) OR TITLE-ABS-KEY (Computer-Assisted) OR TITLE-ABS-KEY (Computerized Health Record*) OR TITLE-ABS-KEY (Electronic Health Record*) OR TITLE-ABS-KEY (electronic information system) OR TITLE-ABS-KEY (Health Communication) OR TITLE-ABS-KEY (Health Informatic*) OR TITLE-ABS-KEY (Information Technology) OR TITLE-ABS-KEY (Information Technology Personnel) OR TITLE-ABS-KEY (interactive digital game) OR TITLE-ABS-KEY (Internet) OR TITLE-ABS-KEY (Mobile health) OR TITLE-ABS-KEY (Occupational Health Service*) OR TITLE-ABS-KEY (online) OR TITLE-ABS-KEY (on-line) OR TITLE-ABS-KEY (personal digital assistant) OR TITLE-ABS-KEY (Public Health Informatic*) OR TITLE-ABS-KEY (Remote Consultation*) OR TITLE-ABS-KEY (remote monitor*) OR TITLE-ABS-KEY (Smartphone) OR TITLE-ABS-KEY (Telehealth) OR TITLE-ABS-KEY (Tele-health) OR TITLE-ABS-KEY (Telemedicine) OR TITLE-ABS-KEY (Tele-medicine) OR TITLE-ABS-KEY (Telenursing) OR TITLE-ABS-KEY (Tele-nursing) OR TITLE-ABS-KEY (web based) OR TITLE-ABS-KEY (webbased) OR TITLE-ABS-KEY (World Wide Web)-- 5,103,663
3. TITLE-ABS-KEY (qualitative) OR TITLE-ABS-KEY (Experience) OR TITLE-ABS-KEY (Perspective) OR TITLE-ABS-KEY (Perception) OR TITLE-ABS-KEY (Interview*) OR TITLE-ABS-KEY (Focus Group*) OR TITLE-ABS-KEY (Grounded Theory) OR TITLE-ABS-KEY (Phenomenol*) OR TITLE-ABS-KEY (content analysis) OR TITLE-ABS-KEY (Anthropology ) OR TITLE-ABS-KEY (Ethnograph*)
4. AND-- **3,884**
