# Supplementary material for: When Tech Meets Touch: Multistakeholder Perspectives and Implementation Strategies for eHealth in Chronic Kidney Disease: A Systematic Review Using Computational Linguistics
Source: J Nurs Manag. 2026 May 13;2026:7612011. doi: 10.1155/jonm/7612011 (PMC13172292; doi:10.1155/jonm/7612011)
Supplement: Supplementary file 2 — Supporting Information 2 Supporting Information 2: Details of the quality appraisal results. [file JONM-2026-7612011-s002.docx]

# Supplementary file 2 Quality appraisal results

**Quality appraisal results of each study using CASP tool**

| Study ID | 1 | 2 | 3 | 4 | 5 | 6 | 7 | 8 | 9 | 10 | Percentage of Yes |
| --- | --- | --- | --- | --- | --- | --- | --- | --- | --- | --- | --- |
| Abdelhady Osman | Y | Y | Y | Y | Y | Y | Y | Y | Y | Y | 100% |
| Aiyegbusi, Olalekan Lee， 2019 | Y | Y | Y | Y | PY | PY | Y | Y | Y | Y | 80% |
| Alexi Vahlkamp, 2024 | Y | Y | Y | Y | Y | Y | Y | Y | Y | Y | 100% |
| Aminu K Bello, 2017 | Y | Y | Y | Y | Y | U | Y | Y | Y | Y | 90% |
| Andrea K. Viecelli | Y | Y | Y | Y | Y | Y | Y | Y | Y | Y | 100% |
| Birgith Engelst, Grove | Y | Y | Y | Y | Y | Y | Y | Y | Y | Y | 100% |
| Charlotte Nielsen | Y | Y | Y | Y | Y | Y | Y | PY | Y | Y | 90% |
| Cyd Eaton, 2020 | Y | Y | Y | Y | Y | Y | Y | Y | Y | Y | 100% |
| Ditchburn, Jae-Llane, 2017 | Y | Y | Y | Y | Y | U | Y | PY | PY | Y | 70% |
| Esmaeel Toni, 2021 | Y | Y | Y | Y | Y | Y | Y | Y | Y | Y | 100% |
| Ginger Chu, 2023 | Y | Y | Y | Y | Y | Y | Y | Y | Y | Y | 100% |
| Hongxia Shen | Y | Y | Y | Y | Y | Y | Y | Y | Y | Y | 100% |
| Jaimon T, Kelly | Y | Y | Y | Y | Y | Y | Y | Y | Y | Y | 100% |
| Jessica Dawson, 2021 | Y | Y | Y | U | PY | U | Y | PY | Y | Y | 60% |
| Keren Ladin | Y | Y | Y | Y | Y | Y | Y | Y | Y | Y | 100% |
| Louise Birk Suder, 2023 | Y | Y | Y | Y | Y | U | Y | Y | Y | Y | 90% |
| Maoliosa Donald,2022 | Y | Y | Y | Y | Y | PY | Y | Y | Y | Y | 90% |
| Molly M. Warner, 2019 | Y | Y | Y | Y | Y | Y | Y | Y | Y | Y | 100% |
| Rachael C Walker, 2020 C | Y | Y | Y | Y | Y | Y | Y | Y | Y | Y | 100% |
| Rachael C Walker, 2020 P | Y | Y | Y | Y | Y | Y | Y | Y | Y | Y | 100% |
| Raquel Scofano, 2022 | Y | U | Y | Y | Y | Y | Y | PY | PY | Y | 70% |
| Suad Esayed, 2025 | Y | Y | Y | Y | Y | Y | Y | Y | Y | Y | 100% |
| Trenton M, Haltom | Y | Y | Y | Y | Y | Y | Y | Y | Y | Y | 100% |
| Percentage of Yes | 100% | 95.65% | 100% | 95.65% | 91.30% | 73.91% | 100% | 82.61% | 91.30% | 100% |  |

**‘Y’=Yes, ‘N’=No, ‘PY’=partial Yes, ‘U’=unclear.**

1. ***Clear statement of aims:*** Was there a clear statement of the aims of the research? What was the goal of the research? Why it was thought important? Its relevance?

2. ***Appropriate qualitative methodology:*** Is a qualitative methodology appropriate? If the research seeks to interpret or illuminate the actions and/or subjective experiences of research participants? Is qualitative research the right methodology for addressing the research goal?

3. ***Research design appropriate to address the aims:*** Was the research design appropriate to address the aims of the research? If the researcher has justified the research design (e.g. have they discussed how they decided which method to use)?

4. ***Appropriate recruitment strategy:*** Was the recruitment strategy appropriate to the aims of the research? If the researcher has explained how the participants were selected? If they explained why the participants they selected were the most appropriate to provide access to the type of knowledge sought by the study If there are any discussions around recruitment (e.g. why some people chose not to take part)

5. ***Data collection:*** Was the data collected in a way that addressed the research issue? If the setting for the data collection was justified? If it is clear how data were collected (e.g. focus group, semi-structured interview etc.)? If the researcher has justified the methods chosen? If the researcher has made the methods explicit (e.g. for interview method, is there an indication of how interviews are conducted, or did they use a topic guide)? If methods were modified during the study. If so, has the researcher explained how and why? If the form of data is clear (e.g. tape recordings, video material, notes etc.)? If the researcher has discussed saturation of data?

6. ***Relationship between researcher and participants:*** Has the relationship between researcher and participants been adequately considered? If the researcher critically examined their own role, potential bias and influence during (a) formulation of the research questions (b) data collection, including sample recruitment and choice of location. How the researcher responded to events during the study and whether they considered the implications of any changes in the research design?

7. ***Ethical issues:*** Have ethical issues been taken into consideration? If there are sufficient details of how the research was explained to participants for the reader to assess whether ethical standards were maintained? If the researcher has discussed issues raised by the study (e.g. issues around informed consent or confidentiality or how they have handled the effects of the study on the participants during and after the study)? If approval has been sought from the ethics committee?

8. ***Data analysis rigor:*** Was the data analysis sufficiently rigorous? If there is an in-depth description of the analysis process? If thematic analysis is used?. If so, is it clear how the categories/themes were derived from the data? Whether the researcher explains how the data presented were selected from the original sample to demonstrate the analysis process? If sufficient data are presented to support the findings? To what extent contradictory data are taken into account Whether the researcher critically examined their own role, potential bias and influence during data analysis and selection of data for presentation?

9. ***Clear statement of findings:*** Is there a clear statement of findings? If the findings are explicit? If there is adequate discussion of the evidence both for and against the researcher’s arguments? If the researcher has discussed the credibility of their findings (e.g. triangulation, respondent validation, more than one analyst)? If the findings are discussed in relation to the original research question?

10. ***Value of study:*** How valuable is the research? If the researcher discusses the contribution the study makes to existing knowledge or understanding (e.g. do they consider the findings in relation to current practice or policy, or relevant research-based literature)? If they identify new areas where research is necessary? If the researchers have discussed whether or how the findings can be transferred to other populations or considered other ways the research may be used?
