# Supplementary material for: When Tech Meets Touch: Multistakeholder Perspectives and Implementation Strategies for eHealth in Chronic Kidney Disease: A Systematic Review Using Computational Linguistics
Source: J Nurs Manag. 2026 May 13;2026:7612011. doi: 10.1155/jonm/7612011 (PMC13172292; doi:10.1155/jonm/7612011)
Supplement: Supplementary file 3 — Supporting Information 3 Supporting Information 3: Details of CERQual Evidence Profile. [file JONM-2026-7612011-s003.docx]

# Supplementary file 3 CERQual Evidence Profile detailed

**CERQual Evidence Profile**

| **Main category of concept** | **Number of studies contributing to the review finding** | **Methodological limitations** | | **Coherence** | **Adequacy** | **Relevance** | **CERQual assessment of confidence in the evidence** | **Explanation of CERQual assessment** |
| --- | --- | --- | --- | --- | --- | --- | --- | --- |
|  |  | **General condition** | **Detailed explanation** |  |  |  |  |  |
| **Accessibility** | **14 studies** |  | **No or very minor concerns for none insufficient domains (proportion not choose "yes" is ＞20%) in CASP:** | **No or very minor concerns – strong fit between findings and data, with no significant contradictory examples** | **Moderate concerns- one statement with only one reference in the core section of stakeholders (nurse).** | **No or very minor concerns regarding relevance—all studies were** | **Moderate confidence** | **Moderate concerns regarding the adequacy. This concerns will lower our confidence.** |
| **Communication Dynamics** | **15 studies** |  | **No or very minor concerns for none insufficient domains (proportion not choose "yes" is ＞20%) in CASP:** | **No or very minor concerns – strong fit between findings and data, with no significant contradictory examples** | **No or very minor concerns –all statement have more than only one reference in the core section of stakeholders.** | **No or very minor concerns regarding relevance—all studies were** | **High confidence.** | **All domain of CERQual Evidence Profile have been rated as No or very minor concerns. Thus, we will not lower our confidence.** |
| **Workflow Integration** | **15 studies** |  | **Minor concerns for 2 insufficient domains (proportion not choose "yes" is ＞20%) in CASP:**  **Relationship between researcher and participants [20.00%]**  **And Data analysis rigor [20.00%]**  **However, these most insufficient domains nearly get a partial yes option. The Methodological limitations could be rated as ‘minor concerns’.** | **No or very minor concerns – strong fit between findings and data, with no significant contradictory examples** | **No or very minor concerns –all statement have more than only one reference in the core section of stakeholders.** | **No or very minor concerns regarding relevance—all studies were** | **High confidence.** | **All domain of CERQual Evidence Profile have been rated as Minor concerns or No or very minor concerns. Thus, we will not lower our confidence.** |
| **Patient Empowerment** | **13 studies** |  | **Minor concerns for 1 insufficient domains (proportion not choose "yes" is ＞20%) in CASP:**  **Relationship between researcher and participants [38.46%]**  **However, these most insufficient domains nearly get a partial yes option. The Methodological limitations could be rated as ‘minor concerns’.** | **No or very minor concerns – strong fit between findings and data, with no significant contradictory examples** | **No or very minor concerns –all statement have more than only one reference in the core section of stakeholders.** | **No or very minor concerns regarding relevance—all studies were** | **High confidence.** | **All domain of CERQual Evidence Profile have been rated as Minor concerns or No or very minor concerns. Thus, we will not lower our confidence.** |
| **Clinical Effectiveness** | **20 studies** |  | **Minor concerns for 1 insufficient domains (proportion not choose "yes" is ＞20%) in CASP:**  **Relationship between researcher and participants [25.00%]**  **However, these most insufficient domains nearly get a partial yes option. The Methodological limitations could be rated as ‘minor concerns’.** | **No or very minor concerns – strong fit between findings and data, with no significant contradictory examples** | **No or very minor concerns –all statement have more than only one reference in the core section of stakeholders.** | **No or very minor concerns regarding relevance—all studies were** | **High confidence.** | **All domain of CERQual Evidence Profile have been rated as Minor concerns or No or very minor concerns. Thus, we will not lower our confidence.** |
