# Supplementary material for: When Tech Meets Touch: Multistakeholder Perspectives and Implementation Strategies for eHealth in Chronic Kidney Disease: A Systematic Review Using Computational Linguistics
Source: J Nurs Manag. 2026 May 13;2026:7612011. doi: 10.1155/jonm/7612011 (PMC13172292; doi:10.1155/jonm/7612011)
Supplement: Supplementary file 4 — Supporting Information 4 Supporting Information 4: Details of CFIR–ERIC strategies. [file JONM-2026-7612011-s004.docx]

# Supplementary file 4 CFIR-ERIC-strategies

| ERIC Strategies | Cumulative Percent | Evidence Strength & Quality | Relative advantage | Adaptability | Complexity | Design Quality & Packaging | Implementation Climate | Compatibility | Available Resources | Access to knowledge & information | Self-efficacy | Executing |
| --- | --- | --- | --- | --- | --- | --- | --- | --- | --- | --- | --- | --- |
| Identify and prepare champions | **283%** | 41% | 45% | 23% | 30% | 15% | 37% | 21% | 4% | 24% | 30% | 14% |
| Promote adaptability | **281%** | 3% | 24% | **73%** | 40% | 48% | 15% | 45% | 4% | 7% | 11% | 10% |
| Assess for readiness and identify barriers and facilitators | **253%** | 13% | 24% | 31% | 30% | 7% | **52%** | 34% | 13% | 7% | 11% | 31% |
| Conduct educational meetings | **241%** | 47% | 24% | 12% | 13% | 22% | 15% | 10% | 0% | **79%** | 15% | 3% |
| Capture and share local knowledge | **232%** | 25% | 17% | 35% | 27% | 15% | 15% | 14% | 22% | 31% | 19% | 14% |
| Create a learning collaborative | **222%** | 16% | 7% | 23% | 33% | 7% | 19% | 14% | 9% | 45% | 30% | 21% |
| Conduct local consensus discussions | **205%** | 41% | 24% | 31% | 7% | 26% | 19% | 41% | 0% | 10% | 0% | 7% |
| Conduct cyclical small tests of change | **203%** | 3% | 31% | 23% | 37% | 11% | 11% | 38% | 13% | 3% | 26% | 7% |
| Develop educational materials | **192%** | 28% | 14% | 12% | 13% | 33% | 0% | 3% | 4% | **59%** | 19% | 7% |
| Tailor strategies | **186%** | 6% | 17% | 35% | 27% | 15% | 19% | 38% | 9% | 0% | 11% | 10% |
| Identify early adopters | **180%** | 22% | 17% | 27% | 20% | 11% | 30% | 10% | 0% | 10% | 19% | 14% |
| Conduct ongoing training | **176%** | 6% | 3% | 0% | 37% | 4% | 11% | 0% | 9% | 38% | 41% | 28% |
| Facilitation | **172%** | 0% | 10% | 27% | 20% | 7% | 22% | 24% | 4% | 10% | 22% | 24% |
| Conduct educational outreach visits | **149%** | 34% | 10% | 12% | 7% | 15% | 7% | 0% | 0% | 28% | 22% | 14% |
| Provide ongoing consultation | **148%** | 9% | 3% | 8% | 20% | 7% | 15% | 3% | 0% | 17% | 41% | 24% |
| Model and simulate change | **146%** | 3% | 10% | 19% | 27% | 11% | 19% | 3% | 0% | 7% | 33% | 14% |
| Purposely reexamine the implementation | **144%** | 6% | 7% | 12% | 17% | 22% | 4% | 28% | 4% | 0% | 0% | 45% |
| Conduct local needs assessment | **144%** | 3% | 34% | 35% | 3% | 15% | 26% | 21% | 0% | 3% | 0% | 3% |
| Distribute educational materials | **141%** | 31% | 10% | 12% | 3% | 19% | 0% | 0% | 0% | **55%** | 4% | 7% |
| Develop a formal implementation blueprint | **140%** | 0% | 7% | 8% | 43% | 15% | 7% | 3% | 4% | 14% | 11% | 28% |
| Visit other sites | **139%** | 13% | 21% | 19% | 3% | 4% | 15% | 10% | 9% | 14% | 15% | 17% |
| Inform local opinion leaders | **137%** | 38% | 28% | 15% | 13% | 19% | 7% | 3% | 0% | 7% | 4% | 3% |
| Organize clinician implementation team meetings | **131%** | 3% | 14% | 8% | 20% | 4% | 11% | 14% | 9% | 14% | 11% | 24% |
| Alter incentive/allowance structures | **131%** | 3% | 28% | 0% | 7% | 0% | 44% | 10% | 17% | 0% | 4% | 17% |
| Provide local technical assistance | **119%** | 3% | 0% | 4% | 17% | 4% | 0% | 14% | 0% | 24% | 22% | 31% |
| Use an implementation adviser | **118%** | 6% | 7% | 8% | 10% | 15% | 7% | 10% | 13% | 14% | 7% | 21% |
| Access new funding | **106%** | 3% | 10% | 0% | 3% | 4% | 0% | 3% | **78%** | 0% | 0% | 3% |
| Stage implementation scale up | **99%** | 3% | 10% | 0% | 30% | 4% | 4% | 10% | 13% | 3% | 15% | 7% |
| Build a coalition | **95%** | 6% | 14% | 15% | 0% | 0% | 19% | 21% | 17% | 3% | 0% | 0% |
| Audit and provide feedback | **95%** | 13% | 10% | 4% | 3% | 4% | 11% | 7% | 0% | 3% | 22% | 17% |
| Develop and implement tools for quality monitoring | **91%** | 6% | 7% | 0% | 7% | 30% | 4% | 3% | 0% | 0% | 4% | 31% |
| Shadow other experts | **90%** | 3% | 3% | 12% | 7% | 4% | 4% | 3% | 0% | 21% | 33% | 0% |
| Make training dynamic | **89%** | 6% | 0% | 0% | 10% | 11% | 4% | 3% | 0% | 10% | 41% | 3% |
| Fund and contract for clinical innovation | **88%** | 0% | 14% | 0% | 3% | 4% | 7% | 10% | 39% | 3% | 4% | 3% |
| Use advisory boards and workgroups | **79%** | 9% | 10% | 4% | 0% | 19% | 11% | 3% | 4% | 0% | 7% | 10% |
| Involve patients/consumers and family members | **78%** | 13% | 3% | 8% | 0% | 19% | 15% | 10% | 0% | 3% | 4% | 3% |
| Facilitate relay of clinical data to providers | **70%** | 6% | 10% | 4% | 3% | 7% | 7% | 3% | 0% | 10% | 7% | 10% |
| Change physical structure and equipment | **69%** | 0% | 3% | 0% | 3% | 0% | 4% | 7% | 48% | 0% | 0% | 3% |
| Obtain and use patients/consumers and family feedback | **68%** | 6% | 7% | 4% | 0% | 30% | 7% | 10% | 0% | 0% | 4% | 0% |
| Develop and organize quality monitoring systems | **59%** | 3% | 3% | 4% | 10% | 0% | 7% | 3% | 0% | 0% | 7% | 21% |
| Recruit, designate and train for leadership | **58%** | 3% | 3% | 0% | 7% | 0% | 26% | 0% | 4% | 3% | 4% | 7% |
| Provide clinical supervision | **56%** | 3% | 0% | 0% | 7% | 0% | 4% | 10% | 0% | 17% | 11% | 3% |
| Develop academic partnerships | **55%** | 25% | 0% | 0% | 0% | 4% | 4% | 0% | 4% | 10% | 7% | 0% |
| Involve executive boards | **52%** | 6% | 3% | 0% | 0% | 4% | 11% | 3% | 17% | 0% | 0% | 7% |
| Use train the trainer strategies | **51%** | 3% | 0% | 0% | 7% | 0% | 4% | 0% | 9% | 10% | 15% | 3% |
| Centralize technical assistance | 49% | 3% | 0% | 0% | 10% | 4% | 0% | 10% | 0% | 3% | 11% | 7% |
| Increase demand | 47% | 0% | 24% | 4% | 3% | 4% | 7% | 0% | 4% | 0% | 0% | 0% |
| Work with educational institutions | 45% | 16% | 3% | 0% | 0% | 11% | 0% | 3% | 4% | 7% | 0% | 0% |
| Revise professional roles | 44% | 0% | 0% | 4% | 3% | 0% | 7% | 10% | 9% | 3% | 0% | 7% |
| Develop resource sharing agreements | 44% | 0% | 7% | 0% | 0% | 0% | 0% | 3% | 26% | 3% | 4% | 0% |
| Intervene with patients/consumers to enhance uptake & adherence | 39% | 3% | 7% | 8% | 3% | 7% | 4% | 3% | 0% | 0% | 0% | 3% |
| Use data experts | 39% | 6% | 10% | 8% | 3% | 4% | 4% | 0% | 0% | 0% | 4% | 0% |
| Promote network weaving | 37% | 0% | 3% | 4% | 0% | 0% | 7% | 0% | 9% | 10% | 4% | 0% |
| Obtain formal commitments | 37% | 0% | 7% | 0% | 0% | 0% | 4% | 0% | 13% | 0% | 0% | 14% |
| Make billing easier | 36% | 0% | 0% | 4% | 3% | 0% | 4% | 0% | 22% | 0% | 0% | 3% |
| Create new clinical teams | 36% | 0% | 7% | 0% | 3% | 0% | 0% | 7% | 4% | 0% | 7% | 7% |
| Mandate change | 36% | 0% | 7% | 0% | 7% | 0% | 15% | 3% | 0% | 0% | 4% | 0% |
| Place innovation on fee for service lists/formularies | 31% | 6% | 3% | 0% | 0% | 0% | 0% | 3% | 17% | 0% | 0% | 0% |
| Alter patient/consumer fees | 25% | 0% | 0% | 0% | 0% | 0% | 4% | 0% | 22% | 0% | 0% | 0% |
| Create or change credentialing and/or licensure standards | 22% | 3% | 3% | 4% | 0% | 0% | 4% | 0% | 4% | 0% | 0% | 3% |
| Use other payment schemes | 22% | 0% | 0% | 0% | 0% | 0% | 0% | 0% | 22% | 0% | 0% | 0% |
| Change liability laws | 18% | 0% | 3% | 0% | 0% | 0% | 7% | 7% | 0% | 0% | 0% | 0% |
| Develop an implementation glossary | 17% | 0% | 0% | 0% | 3% | 4% | 0% | 0% | 0% | 7% | 0% | 3% |
| Use mass media | 17% | 6% | 0% | 0% | 0% | 0% | 4% | 3% | 0% | 3% | 0% | 0% |
| Change record system | 15% | 0% | 0% | 0% | 0% | 0% | 0% | 0% | 4% | 0% | 4% | 7% |
| Change service sites | 11% | 3% | 0% | 0% | 0% | 0% | 0% | 3% | 4% | 0% | 0% | 0% |
| Prepare patients/consumers to be active participants | 11% | 0% | 0% | 0% | 0% | 0% | 7% | 3% | 0% | 0% | 0% | 0% |
| Use data warehousing techniques | 11% | 3% | 0% | 8% | 0% | 0% | 0% | 0% | 0% | 0% | 0% | 0% |
| Use capitated payments | 9% | 0% | 0% | 0% | 0% | 0% | 0% | 0% | 9% | 0% | 0% | 0% |
| Develop disincentives | 7% | 0% | 3% | 0% | 0% | 0% | 4% | 0% | 0% | 0% | 0% | 0% |
| Remind clinicians | 7% | 0% | 0% | 0% | 0% | 0% | 0% | 0% | 0% | 3% | 0% | 3% |
| Start a dissemination organization | 4% | 0% | 0% | 0% | 0% | 4% | 0% | 0% | 0% | 0% | 0% | 0% |
| Change accreditation or membership reqs | 3% | 0% | 3% | 0% | 0% | 0% | 0% | 0% | 0% | 0% | 0% | 0% |
